# A network pharmacology-based approach to explore the active ingredients and molecular mechanism of Lei-gong-gen formula granule on a spontaneously hypertensive rat model

**DOI:** 10.1186/s13020-021-00507-1

**Published:** 2021-10-09

**Authors:** Qiaofeng Li, Taijin Lan, Songhua He, Weiwei Chen, Xiaolan Li, Weiquan Zhang, Ying Liu, Qiuping Zhang, Xin Chen, Yaoyao Han, Zhiheng Su, Dan Zhu, Hongwei Guo

**Affiliations:** 1 Guangxi Key Laboratory of Bioactive Molecules Research and Evaluation & College of Pharmacy, Guangxi Medical University, 22 Shuangyong Road, Nanning, 530021 China; 2 Key Laboratory of Longevity and Aging-related Diseases of Chinese Ministry of Education & Center for Translational Medicine, School of preclinical medicine, Guangxi Medical University, Nanning, 530021 Guangxi China; 3 School of preclinical medicine, Guangxi University of Chinese Medicine, 179 Mingxiu Dong Road, Nanning, 530001 China; 4 Guangxi Institute for Food and Drug Control, 9 Qinghu Road, Nanning, 530021 China; 5 Guangxi Key Laboratory of Regenerative Medicine, Guangxi Medical University, Nanning, 530021 China; 6 International Joint Laboratory on Regeneration of Bone and Soft Tissues, Guangxi Medical University, Guangxi, 530021 China; 7 College of Pharmacy, Guangxi University of Chinese Medicine, 179 Mingxiu Dong Road, Nanning, 530001 China; 8 The First Affiliated Hospital, Guangxi Medical University, 6 Shuangyong Road, Nanning, 530021 China

**Keywords:** *Centella asiatica* (L.) Urb., *Eclipta prostrata* (L.) L., *Smilax glabra* Roxb., Hypertension, Network pharmacology

## Abstract

**Background:**

Lei-gong-gen formula granule (LFG) is a folk prescription derived from Zhuang nationality, the largest ethnic minority among 56 nationalities in China. It consists of three herbs, namely *Eclipta prostrata* (L.) L., *Smilax glabra* Roxb, and *Centella asiatica* (L.) Urb. It has been widely used as health protection tea for hundreds of years to prevent hypertension in Guangxi Zhuang Autonomous Region. The purpose of this study is to validate the antihypertensive effect of LFG on the spontaneously hypertensive rat (SHR) model, and to further identify the effective components and anti-hypertension mechanism of LFG.

**Methods:**

The effects of LFG on blood pressure, body weight, and heart rate were investigated *in vivo* using the SHR model. The levels of NO, ANG II, and ET-1 in the serum were measured, and pathological changes in the heart were examined by H&E staining. The main active components of LFG, their corresponding targets, and hypertension associated pathways were discerned through network pharmacology analysis based on the Traditional Chinese Medicine Systems Pharmacology (TCMSP), Traditional Chinese Medicine Integrated Database (TCMID), and the Bioinformatics Analysis Tool for Molecular Mechanism of Traditional Chinese Medicine (BATMAN-TCM). Then the predicted results were further verified by molecular biology experiments such as RT-qPCR and western blot. Additionally, the potential active compounds were predicted by molecular docking technology, and the chemical constituents of LFG were analyzed and identified by UPLC-QTOF/MS technology. Finally, an *in vitro* assay was performed to investigate the protective effects of potential active compounds against hydrogen peroxide (H_2_O_2_) induced oxidative damage in human umbilical vein endothelial cells (HUVEC).

**Results:**

LFG could effectively reduce blood pressure and increase serum NO content in SHR model. Histological results showed that LFG could ameliorate pathological changes such as cardiac hypertrophy and interstitial inflammation. From network pharmacology analysis, 53 candidate active compounds of LFG were collected, which linked to 765 potential targets, and 828 hypertension associated targets were retrieved, from which 12 overlapped targets both related to candidate active compounds from LFG and hypertension were screened and used as the potential targets of LFG on antihypertensive effect. The molecular biology experiments of the 12 overlapped targets showed that LFG could upregulate the mRNA and protein expressions of NOS3 and proto-oncogene tyrosine-protein kinase SRC (SRC) in the thoracic aorta. Pathway enrichment analysis showed that the PI3K-AKT signaling pathway was closely related to the expression of NOS3 and SRC. Moreover, western blot results showed that LFG significantly increased the protein expression levels of PI3K and phosphorylated AKT in SHR model, suggesting that LFG may active the PI3K-AKT signaling pathway to decrease hypertension. Molecular docking study further supported that p-hydroxybenzoic acid, cedar acid, shikimic acid, salicylic acid, nicotinic acid, linalool, and histidine can be well binding with NOS3, SRC, PI3K, and AKT. UPLC-QTOF/MS analysis confirmed that p-hydroxybenzoic acid, shikimic acid, salicylic acid, and nicotinic acid existed in LFG. Pre-treatment of HUVEC with nicotinic acid could alleviate the effect on cell viability induced by H_2_O_2_ and increase the NO level in cell supernatants.

**Conclusions:**

LFG can reduce the blood pressure in SHR model, which might be attributed to increasing the NO level in serum for promoting vasodilation via upregulating SRC expression level and activating the PI3K-AKT-NOS3 signaling pathway. Nicotinic acid might be the potential compound for LFG antihypertensive effect.

**Supplementary Information:**

The online version contains supplementary material available at 10.1186/s13020-021-00507-1.

## Introduction

Hypertension is one of the most common cardiovascular diseases, which can cause damage to the heart, brain, kidney, and other important organs [1]. It is an important risk factor for the death of the global population and poses a serious threat to patient safety and quality of care. In China, large-scale population surveys of hypertension have reported a rapidly increasing prevalence of hypertension, from 5.1% to 1959 to 27.8% in 2014 [2]. In Europe, Canada, and the United States, the prevalence varied from 27 to 55% [3]. Hypertension is a chronic disease, and there is no complete cure for high blood pressure. Currently, drug treatment is mainly used to control blood pressure and bring it down to a normal range in clinical practice. Although the existing antihypertensive drugs can effectively control blood pressure in the short term, it is prone to develop adverse effects for long term medication, which greatly affects their treatment compliance [4]. Therefore, the development of drugs, which can effectively lower blood pressure with fewer adverse reactions, is still the current research direction. Traditional Chinese Medicine (TCM) has accumulated much experience in the treatment of hypertension [5]. TCM has the characteristics of multi-component, multi-target, and multi-pathways, especially has a certain effect in improving the complications of hypertension [6]. Thus, screening high-efficiency antihypertensive drugs with fewer side effects based on Chinese medicine formula has attracted considerable attention.

Lei-gong-gen formula granule (LFG) is an effective antihypertensive traditional Chinese folk prescription, and residents of Guangxi Zhuang Autonomous Region have soaked it in wine or used as tea for many years [7]. Epidemiological data showed that the prevalence of hypertension among residents was 13.70%, which was significantly lower than the national average (25.2%) [8]. It is speculated that the lower incidence of hypertension of the local people may be related to their long-term use of this formula. LFG is composed of three herbs, containing *Centella asiatica* (L.) Urb. (Ji-xue-cao, also known as Lei-gong-gen in Chinese medicine), *Eclipta prostrata* (L.) L. (Mo-han-lian), *and Smilax glabra* Roxb. (Tu-fu-ling). *Centella asiatica* (L.) Urb. is a tropical medicinal plant belonging to the family *Apiaceae* (Umbelliferae). It is widely distributed in many regions of the world, especially in Asian countries, including India, China, Indonesia and Nepal. *Centella asiatica* (L.) Urb. was reported to exhibit cardioprotective, antihypertensive, antioxidant, and anti-inflammatory activities [9]. Recent clinical study from Indonesia has shown that gotu kola (*Centella asiatica* L.) tea can decrease blood pressure of hypertension patients [10]. Asiatic acid could reduce blood pressure and improve vascular function by restoring the expression of endothelial NO synthase (eNOS) and p47phox, thereby alleviating cardiovascular remodeling by restoring iNOS/eNOS expression in L-NAME hypertensive rats [11]. *Eclipta prostrata* (L.) L. is an annual herb of the Asteraceae family, native to China, Japan, and India [12]. *Eclipta prostrata* (L.) L. was reported to have significant pharmacological features, such as antioxidant, anti-inflammatory, and hypotensive activities [13]. *Smilax glabra* Roxb. is the rhizome of the Liliaceae plant [14]. Pharmacological studies demonstrated that the flavonoids from *Smilax glabra* Roxb. exhibited anti-cardiac hypertrophy and anti-hypertensive effects on renovascular hypertension rats by regulating atrial natriuretic peptide levels [15].

Chinese herbal formulae contain a large number of compounds and it is too complex to be analyzed by traditional experimental methods based on the paradigm of “One gene, one drug, one disease” [16]. Along with the rapid development of bioinformatics, a newly emerging network pharmacology that uses a mathematical and computable representation of various connections between herbal formulae and diseases has been proposed [17]. The holistic philosophy is the main feature of TCM, which meets the critical idea of network pharmacology [18]. Focusing on studying drugs, network pharmacology provides new ideas, especially for TCM research, which is based on a complex system. It has provided new scientific and technological support for the rational clinical use of drugs and drug development [19]. Accumulating evidence shows that network pharmacology can clarify the potential mechanisms of multi-component and multi-target agents by analyzing various complex and multi-level interaction networks [20].

**Fig. 1:**
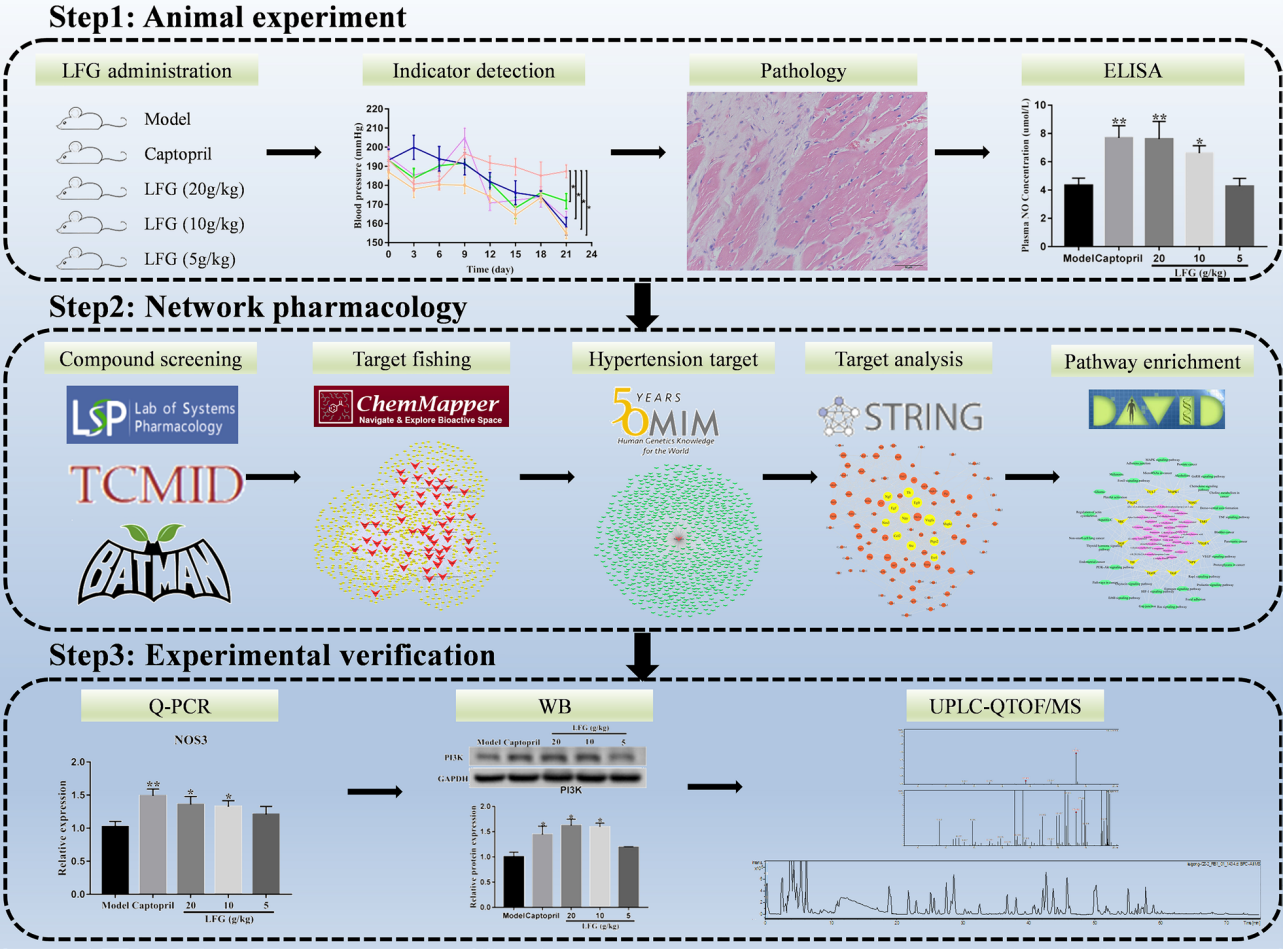
Work flow of the study

In this study, the active ingredients, corresponding targets, and pharmacological mechanisms of LFG antihypertensive effect were investigated by integrating network pharmacology prediction and molecular biology experimental validations. As shown in Fig. 1, the entire study consists of three steps. Step 1: animal experiments. The spontaneously hypertensive rat (SHR) model was used to observe the antihypertensive effect of LFG. Step 2: network pharmacology analysis. The effective ingredients, corresponding targets, and hypertension associated pathways were predicted through public databases. The protein-protein interaction (PPI) networks of LFG-related targets and hypertension-related targets were constructed, and the core targets were identified through topological analysis. Step 3: experimental verification. Based on the SHR model, the predicted results were verified by molecular biology methods such as RT-qPCR and western blot. The potential active ingredients were predicted by molecular docking technology, and the LFG chemical constituents were analyzed and identified by Ultra performance liquid chromatography/quadrupole time-of-flight mass spectrometry (UPLC-QTOF/MS) technology. The ingredients which confirmed by UPLC-QTOF/MS were used to investigate the protective effects against H_2_O_2_ induced oxidative damage in human umbilical vein endothelial cells (HUVEC). The findings of this study may contribute to the clarification of active ingredients and underlying mechanisms of LFG and provide an accurate and reasonable reference for the clinical application of LFG.

## Materials and methods

### Drugs and reagents

Lei-gong-gen formula granule (LFG) was purchased from Guangxi Institute of Chinese Medicine and Pharmaceutical Science (Guangxi, China, lot No. 20,170,210). The authentication of three dried herbal drugs (*Centella asiatica* (L.) Urb., *Eclipta prostrata* (L.) L., *Smilax glabra* Roxb.) has been specified, and the preparation of LFG was shown in our previous research [21, 22]. Captopril was obtained from Teyi Pharmaceutical Group Co., Ltd (Guangdong, China, lot No. 20,170,501). p-hydroxybenzoic acid (lot No. H108509), shikimic acid (lot No. S107143), salicylic acid (lot No. S118533), and nicotinic acid (lot No. N103654) were purchased from Aladdin Reagent Co., Ltd. (Shanghai, China).

The Nitric oxide (NO) assay kit was obtained from Nanjing Jiancheng Bioengineering Institute (Nanjing, China, Cat. A013-2 and A012-1). Rat angiotensin II (ANG II, Cat. CSB-E04494r) and rat endothelin 1 (ET-1, Cat. CSB-E06979r) ELISA kits were obtained from CUSABIO (Wuhan, China). Trizol reagent (Cat. 15,596,026), cDNA Reverse Transcription Kit (Cat. 4368814), SYBR Green Master Mix (Cat. A25742), Pierce Protease and Phosphatase Inhibitor Mini Tablets (Cat. A32961), RIPA buffer (Cat. 89900), Prestained Protein Ladder (Cat. 26616), Tween 20 (Cat. 85113), Chemiluminescent Substrate (Cat. 24580) were purchased from Thermo Fisher Scientific (Waltham, USA). The Bicinchoninic acid (BCA) Protein Assay Kit (No. P0011) was purchased from Beyotime Biotechnology Co., Ltd. (Shanghai, China).

GAPDH (14C10) Rabbit mAb (Cat. 2118), SRC (36D10) Rabbit mAb (Cat. 2109), PI3 Kinase p110α (C73F8) Rabbit mAb (Cat. 4249), AKT Antibody (Cat. 9272), Phospho-AKT (Ser473) (D9E) XP Rabbit mAb (Cat. 4060) and Anti-Rabbit IgG HRP-linked antibodies (Cat.7074) were purchased from Cell Signaling Technology (Danvers, USA). Anti-NOS3 antibody [EPR19296] (Cat. Ab199956) was purchased from Abcam (Shanghai, China).

Methanol (HPLC grade) was purchased from Sinopharm Chemical Reagent Co., Ltd. (Shanghai, China). Formic acid (HPLC grade) was purchased from Aladdin Reagent Co., Ltd. (Shanghai, China). The reference standard of shikimic acid (lot No. C11015240) was purchased from Shanghai Macklin Biochemical Co., Ltd. The reference standard of isoengelitin (lot No. ZW190519-14) was purchased from Stanford Analytical Chemicals Inc. The reference standard of 4-dicaffeoylquinic acid (lot No. 34770010) was purchased from ANPEL Laboratory Technologies (Shanghai) Inc. The reference standard of p-hydroxybenzoic acid (lot No. H1926195) was purchased from Shanghai Aladdin Reagent Co., Ltd. The reference standards of engeletin (lot No. 111906–201102), astilbin (lot No. 111798–201504), ferulic acid (lot No. 11073–201313), luteolin (lot No. 111520–201605), isochlorogenic acid C (lot No. 111894–201102), salicylic acid (lot No. 100106–201104), and nicotinic acid (lot No. 100434–201603) were purchased from National Institutes for Food and Drug Control (Beijing, China). The reference standards of neoastilbin (lot No. DST191209-077), isoastilbin (lot No. DST190922-216), neoisoastilbin (lot No. DST191025-078) were purchased from Chengdu Pufei De Biotech Co., Ltd.

### Animals and experimental design

A total of 40 male Spontaneously hypertensive rats (SHR) (180-220 g), aged 10 weeks, were provided by Beijing Vital River Laboratory Animal Technologies Co. Ltd (Beijing, China, certificate number: SCXK-JING 2016-0006). All animals were maintained under standard laboratory conditions with a regular 12-h light/dark cycle, relative humidity of 50 ± 5%, and the temperature was maintained at 25 ± 2 °C. The experimental procedures were approved by the Institutional Animal Ethics Committee (IAEC), Guangxi Medical University.

The rats were provided with standard rat chow and water *ad libitum*. After an acclimatization period for two weeks, the basic value of blood pressure, body weight, and heart rate were measured. SHRs were randomly assigned to five groups based on the basic value of blood pressure: model group (saline, 10 mL/kg), captopril group (0.03 g/kg), LFG high dose group (20 g/kg), LFG middle dose group (10 g/kg), LFG low dose group (5 g/kg), eight rats per group. Saline served as a negative control and captopril, a proven hypertensive drug [23], as a positive control. LFG and captopril were administered twice a day intragastrically for 3 weeks. The systolic blood pressure, body weight, and heart rate of rats were measured every 3 days. The blood pressure and heart rate were measured using ALC-NIBP non-invasive blood pressure system (Shanghai Alcott Biotech Co., Ltd, China) at the tail artery employing the tail-cuff method, while body weight was measured using an electronic balance (CP214, Ohaus, USA). When the experimental period was complete, rats were anesthetized and blood was collected from the abdominal aorta. The serum samples were obtained after the blood samples were centrifuged. The heart and thoracic aorta were collected respectively for hematoxylin-eosin (H&E) staining and stored at -80ºC for real-time PCR and western blot analysis.

### Determination of serum NO, ANG II, and ET-1 content

The serum NO level in serum was measured by Nitric oxide (NO) assay kit obtained from Nanjing Jiancheng Bioengineering Institute. The serum ANG II and ET-1 contents were measured respectively using commercially available immunoassay ELISA kits for rats.

### Heart histopathological analysis

Heart tissue samples were fixed in 4% paraformaldehyde, dehydrated, and washed. The samples were embedded in paraffin and sliced into 5 μm by a paraffin slicing machine. The sections were dewaxed in xylene and dehydrated in descending grades of ethanol. The sections were stained by hematoxylin for 5 min. After washing, the sections were treated with eosin solution for 2 min for coloration, followed by ethanol dehydration and neutral gum sealing. Finally, the specimens were further observed and photographed under a BX53 microscope (Olympus, Takachiho, Japan).

### Network pharmacology analysis

#### Composite compounds of LFG

LFG consists of three herbs, namely *Centella asiatica* (L.) Urb., *Eclipta prostrata* (L.) L., *Smilax glabra* Roxb. The compounds of LFG were collected using the TCM Systems Pharmacology Database [24] (TCMSP, http://tcmspw.com/tcmsp.php) (accessed 18th January 2018), TCM Integrated Database [25] (TCMID, http://www.megabionet.org/tcmid/) (accessed 18th January 2018), and Bioinformatics Analysis Tool for Molecular mechanism of TCM [26] (BATMAN-TCM, http://bionet.ncpsb.org/batman-tcm/) (accessed 19th January 2018). Candidate compounds were screened according to the criteria of oral bioavailability (OB) > 30% of the pharmacokinetic parameters [27].

#### Targets collection

The SMILES of the candidate compounds in LFG were used as the inputs of target fishing database, obtained from Pubchem database (https://pubchem.ncbi.nlm.nih.gov/). Subsequently, the predicted targets were retrieved from ChemMapper database [28] (http://lilab.ecust.edu.cn/chemmapper/) (accessed 26th January 2018), an online tool based on 3D similarity for targets prediction. The targets associated with hypertension were collected by Text-mined Hypertension, Obesity and Diabetes candidate gene database [29] (T-HOD, http://bws.iis.sinica.edu.tw/THOD/) (accessed 28th January 2018), a tool developed to help trace existing research on three kinds of cardiovascular disease. Furthermore, the overlapped targets both related to the candidate compounds and hypertension were kept for network construction and analysis.

#### Network construction

Network construction was performed as follows: (1) Herb-compound network; (2) Compound-target network; (3) Hypertension-target network; (4) The protein-protein interaction (PPI) network of intersection targets between compounds and hypertension targets.

All the networks were created via Cytoscape (htttps://cytoscape.org/, Version 3.2.1), a general platform for complex network analysis and visualization [30]. The PPI network was constructed by Search Tool for the Retrieval of Interacting Genes/Proteins database [31] (STRING, https://string-db.org/, Version 11.0) with medium confidence 0.400 by default, and then visualized in Cytoscape. The topological parameters of nodes in PPI network were calculated by Network Analyzer, and the nodes whose degree values were twice the average degree values of the network were selected as the hub targets for further analysis.

#### Pathway enrichment

The Database for Annotation, Visualization and Integrated Discovery [32] (DAVID, https://david.ncifcrf.gov/, Version 6.8) was applied for Kyoto Encyclopedia of Genes and Genomes (KEGG) pathway enrichment analysis of the hub targets. *P*-values were set at 0.05 as the cut-off criterion.

### Real-time RT-PCR analysis

Quantitative PCR analysis was performed to examine the mRNA expression levels of predicted hub targets. In brief, total RNA was extracted from thoracic aorta tissue using Trizol reagent, and the quality and purity of RNA were evaluated by Nanodrop2000 (Thermo Fisher, USA). The RNA (2 µg) was reverse-transcribed using the High-Capacity cDNA Reverse Transcription Kit under reaction conditions with 25 °C for 10 min, 37 °C for 120 min and 85 °C for 5 min. Real-time PCR amplification was performed with SYBR Green Master Mix on the 7300 Real-time PCR system (Applied Biosystems) following the manufacturer’s instructions. The relative RNA expression levels were calculated using the ΔΔ cycle threshold method [33], and β-actin was applied to normalize the expression levels of target genes for each sample. Primer sequences were listed in Table 1.

**Table 1:** Primer sequence used for PCR analysis

Gene	Forward	Reverse
NOS3	5’-CAGAGATTGGCATGAGGGACC-3’	5’-TCCACAGTGATGAGGTTGTCC-3’
SRC	5’-CAAGATCACTAGACGGGAATCAG-3’	5’-GTTTCACATTTAGGCCCTTGG-3’
PTGS2	5’-ATCAGAACCGCATTGCCTCT-3’	5’-GCCAGCAATCTGTCTGGTGA-3’
NGF	5’-GTCTGGGCCCAATAAAGGCT-3’	5’-TGTACGCCGATCAAAAACGC-3’
VEGFA	5’-CGTCCTGTGTGCCCCTAATG-3’	5’-TGTGCTGGCTTTGGTGAGGT-3’
EGF	5’-CTACTACAGGACTCGGAAGCAG-3’	5’-GTTGGGGACCAGAAGACACC-3’
EGFR	5’-AGCCGTCCTGTCCAACTATG-3’	5’-TTGCTAAATCGCACAGCACC-3’
ERK2	5’-ATTGGTCAGGACAAGGGCTCA-3’	5’-CCACTACGACCAGAACTGCC-3’
CCL2	5’-TGCAGGTCTCTGTCACGCTTC-3’	5’-TTCTCCAGCCGACTCATTGG-3’
NPY	5’-GCCAGATACTACTCCGCTCTG-3’	5’-GTCTCAGGGCTGGATCTCTTG-3’
ESR1	5’-CAGACAGGGAGCTGGTTCATA-3’	5’-GCACACTCCAGAAGGTGAACT-3’
TH	5’-GTCTCAGAGCAGGATGCCAAG-3’	5’-ATCCTCGATGAGACTCTGTCGC-3’
β-actin	5’-GTCAGGTCATCACTATCGGCAAT-3’	5’-AGAGGTCTTTACGGATGTCAACGT-3’

### Western blot analysis

The protein expression levels of endothelial nitric oxide synthase 3 (NOS3), proto-oncogene tyrosine-protein kinase SRC (SRC), phosphatidylinositol 3 kinase (PI3K), protein kinase B (AKT) and p-AKT were determined by western blot analysis. Total proteins extracted from thoracic aorta by lysis buffer supplemented with phosphatase and protease inhibitors. The protein concentrations were measured by BCA Protein Assay Kit. Protein samples (30 µg) were separated on 10% sodium dodecyl sulphate-polyacrylamide gel electrophoresis (SDS-PAGE) and then transferred to polyvinylidene difluoride (PVDF) membranes. The membranes were blocked for 1 h at 4 °C with 5% nonfat milk, while the phosphorylated proteins were blocked with 5% bovine serum albumin (BSA), and incubated overnight with anti-NOS3 (1:1000), anti-SRC (1:1000), anti-PI3K (1:1000) antibody, anti-AKT (1:1000) antibody, anti-p-AKT (1:1000) antibody and anti-GAPDH (1:5000) antibody, respectively. The membranes were washed 3 times and incubated with secondary antibody (horseradish peroxidase-conjugated, 1:5000 dilution) for 1 h at room temperature. The GAPDH was applied to show equal protein loading for each sample. The bands were visualized by chemiluminescence using MiniChemi 610 Plus (Sage Creation, Beijing), and Image J software (Version 1.43) was used to analyze the intensity of bands.

### Molecular docking

Molecular docking is a computational tool that can predict the interaction energy between receptors and ligands. The binding mode and affinity between targets and compounds were performed using the Surflex-Dock (SFXC) module of Sybyl X2.0 and visualized with PyMOL (Version 1.5.0.3). The structures of the potential active compounds related to the potential targets were downloaded from the Pubchem database and converted into mol2 format by Open Babel GUI (Version 2.2.1). All the crystal structures of the targets were downloaded from Protein Data Bank (PDB, https://www.rcsb.org/), including endothelial nitric oxide synthase (NOS3) (PDB ID: 4D1O), SRC (PDB ID: 1A07), PI3K (PDB ID: 4L23), and AKT (PDB ID: 1H1O). Before docking, the proteins were subjected to the necessary preparation steps (remove water molecules and add hydrogen atoms), and ligands were optimized by the Tripos force field. The total score (pKd) calculated by SFXC was used to determine the affinity of receptors and ligands. The total score greater than 6 was regarded as good protein-ligand binding.

### UPLC-QTOF/MS analysis of LFG

LFG extract was dissolved by ultrasonification in 50% methanol (in water) for 1 h, and filtered through a 0.22 μm syringe filter before analysis.

UPLC analysis conditions were as follows: Separation of the compounds was carried out on a Dionex Ultimate 3000 UPLC system (Thermo Fisher, USA) equipped with a SunFire C18 column (250 mm × 4.6 mm, i.d., 4.6 μm, Waters, USA). The analytical column was maintained at a temperature of 35 °C and the mobile phase was composed of water A (containing 0.1% formic acid) and acetonitrile B. A solvent gradient system was used: 10–10% B for 0–10 min, 10–30% B for 10–50 min, 30–90% B for 50–58 min, 90–90% B for 58–64 min, 90–10% B for 64–65 min and 10–10 %B for 65–75 min. The flow rate was 0.8 mL/min. The injection volume was 10 µL. A DAD detector and a mass spectrometer detector were connected in series behind the column. The outlet of the DAD detector was split into the mass spectrometer detector with a split ratio of 4:1.

MS analysis conditions were as follows: MS analysis was performed on the Bruker definition accurate mass quadrupole time-of-flight (Q-TOF) Impact II mass spectrometer (Bruker Daltonics Inc., GER) equipped with electrospray ionization (ESI) source. The capillary voltage was set at 2500 V, the dry temperature was 200 °C, the dry gas rate was set at 4.0 L/min, the energies for collision induced dissociation (CID) were 10 eV for the precursor ion and 32.5–97.5 eV for fragmentation information. A full-scan mass range of m/z 50-2000 was scanned. Data were acquired in Line Spectra mode.

The contents of shikimic acid, nicotinic acid, p-hydroxybenzoic acid and salicylic acid in LGF were determined by LC-MS/MS. Four reference substances were mixed with methanol, and a series of curve solutions were prepared. Agilent1290-6490 liquid chromatography-mass spectrometry was used for the determination with acetonitrile-0.1% formic acid solution as mobile phase. Shikimic acid and nicotinic acid were determined by Waters SunFire C18 column (250 × 4.6 mm, 5 μm). p-hydroxybenzoic acid and salicylic acid were determined by Agilent Eclipse Plus C18, RRHD column (2.1 × 100 mm, 1.8 μm).

## In vitro experiment verification

### Cell viability assessment

Human umbilical vein endothelial cells (HUVEC) were cultured in endothelial cell growth medium-2 (EGM-2) (LONZA, Walkersville, MD USA, Cat. CC-4176) containing 2% FBS, 0.2 mL hydrocortisone, 2 mL hFGF-B, 0.5 mL VEGF, 0.5 mL R3-IGF-1, 0.5 mL ascorbic acid, 0.5 mL hEGF, 0.5 mL GA-1000 and 0.5 mL heparin. HUVEC (2 × 10^4^ cells/well) were seeded and allowed to adhere in 96-well plates overnight. The cells were pre-treated for 24 h with p-hydroxybenzoic acid (0.1 µM, 1 µM, 10 µM, 100 µM), shikimic acid (0.1 µM, 1 µM, 10 µM, 100 µM), salicylic acid (1 µM, 10 µM, 100 µM, 1000 µM), and nicotinic acid (1 µM, 10 µM, 100 µM, 1000 µM), then H_2_O_2_ with a final concentration of 600 µM was added to stimulate the cells for 6 h. Captopril was used as a positive control. After incubation, the supernatants were removed, washed with PBS, and replaced with fresh medium. The cells were added with 10 µL CCK8 for 2 h in dark, and the absorbance was measured at 450 nm in a microplate reader (Multiskan FC, Thermo Fisher, USA).

### NO release assay

After HUVEC were treated, the supernatant was collected and the level of NO was determined using assay kit according to the protocol described by the manufacturer.

### Statistical analysis

Statistical analysis was performed by SPSS software (Version 22.0). The data were expressed as mean ± SEM. The parameters among different groups were compared by one-way analysis of variance (ANOVA) followed by LSD (equal variances) or Dunnett’s T3 (unequal variances). *P*-value < 0.05 was considered as statistically significant.

## Results

### LFG exerts an anti-hypertensive effect in SHR

**Fig. 2:**
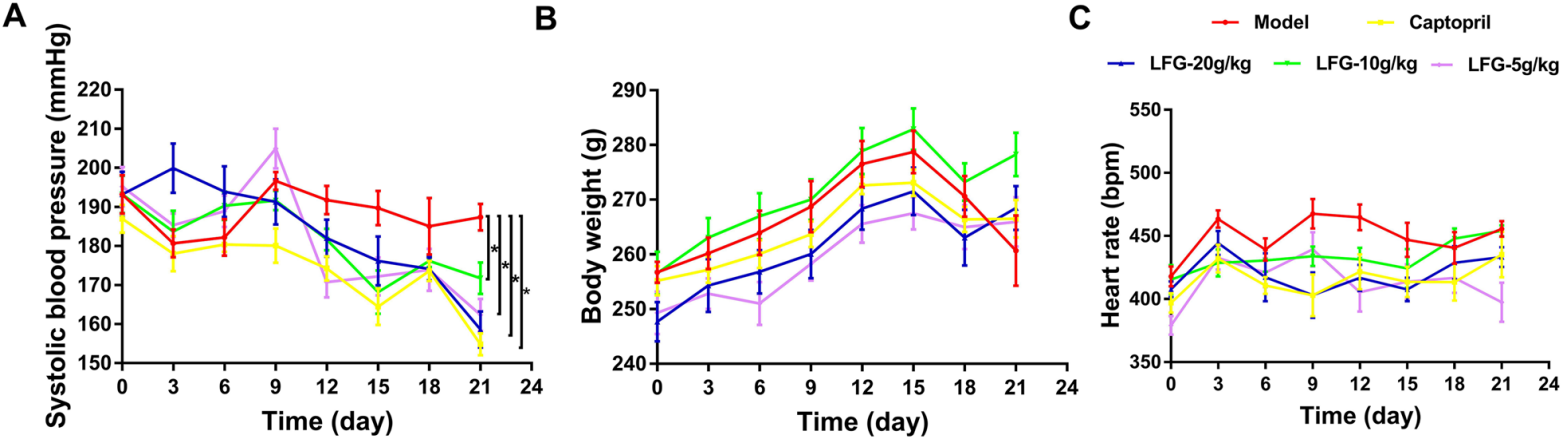
Effect of LFG on blood pressure, body weight and heart rate of SHR. **A** Blood pressure changes; **B** body weight changes; **C** heart rate changes. Compared with the model group, *P < 0.05

To assess the anti-hypertensive effect of LFG, SHRs were treated with different doses of LFG for 3 weeks. As shown in Fig. 2A, the blood pressure of LFG treatment groups was significantly decreased compared to model group (*P* < 0.05). But neither body weight nor heart rate has differences between LFG treatment groups and the model group (*P* > 0.05) (Fig. 2B, C).

### Effect of LFG on serum NO, ANG II and ET-1 levels

**Fig. 3:**
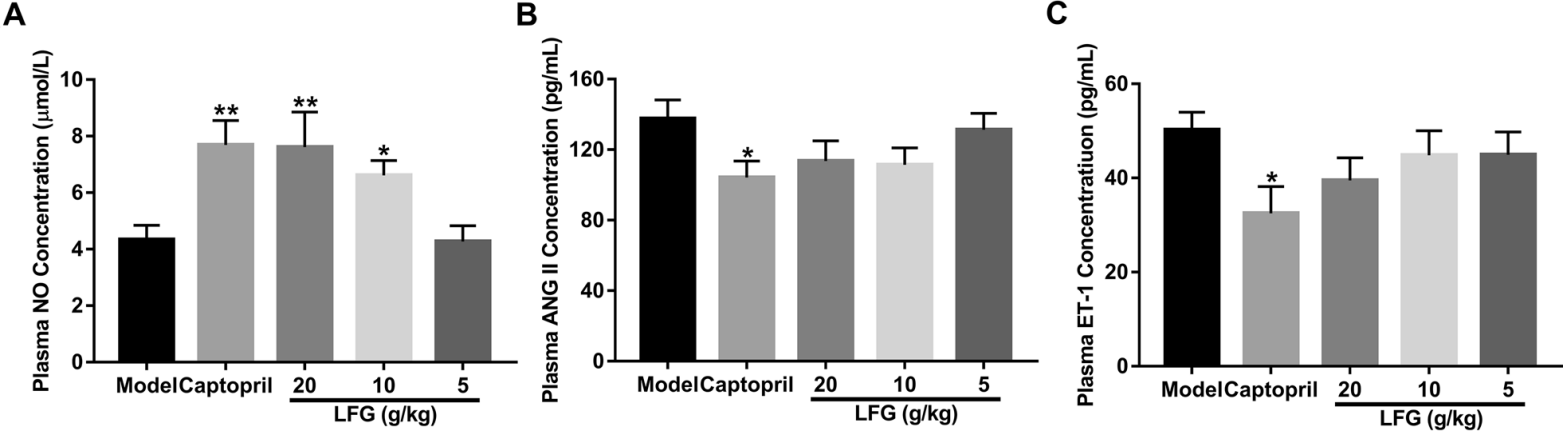
Effect of LFG on **A** NO, **B** Ang II and **C** ET-1 levels in serum of SHR. Compared with model group, * *P* < 0.05, ** *P* < 0.01

Serum NO, ANG II and ET-1 levels of animals from various groups were summarized in Fig. 3. The level of serum NO increased significantly in LFG high-dose group and LFG middle-dose group compared to the model group (*P* < 0.01 or 0.05) (Fig. 3A). However, there was no effect on the contents of ANG II and ET-1 in serum after treatment with different doses of LFG (*P* > 0.05) (Fig. 3B, C).

### Effect of LFG on myocardial morphological change

**Fig. 4:**
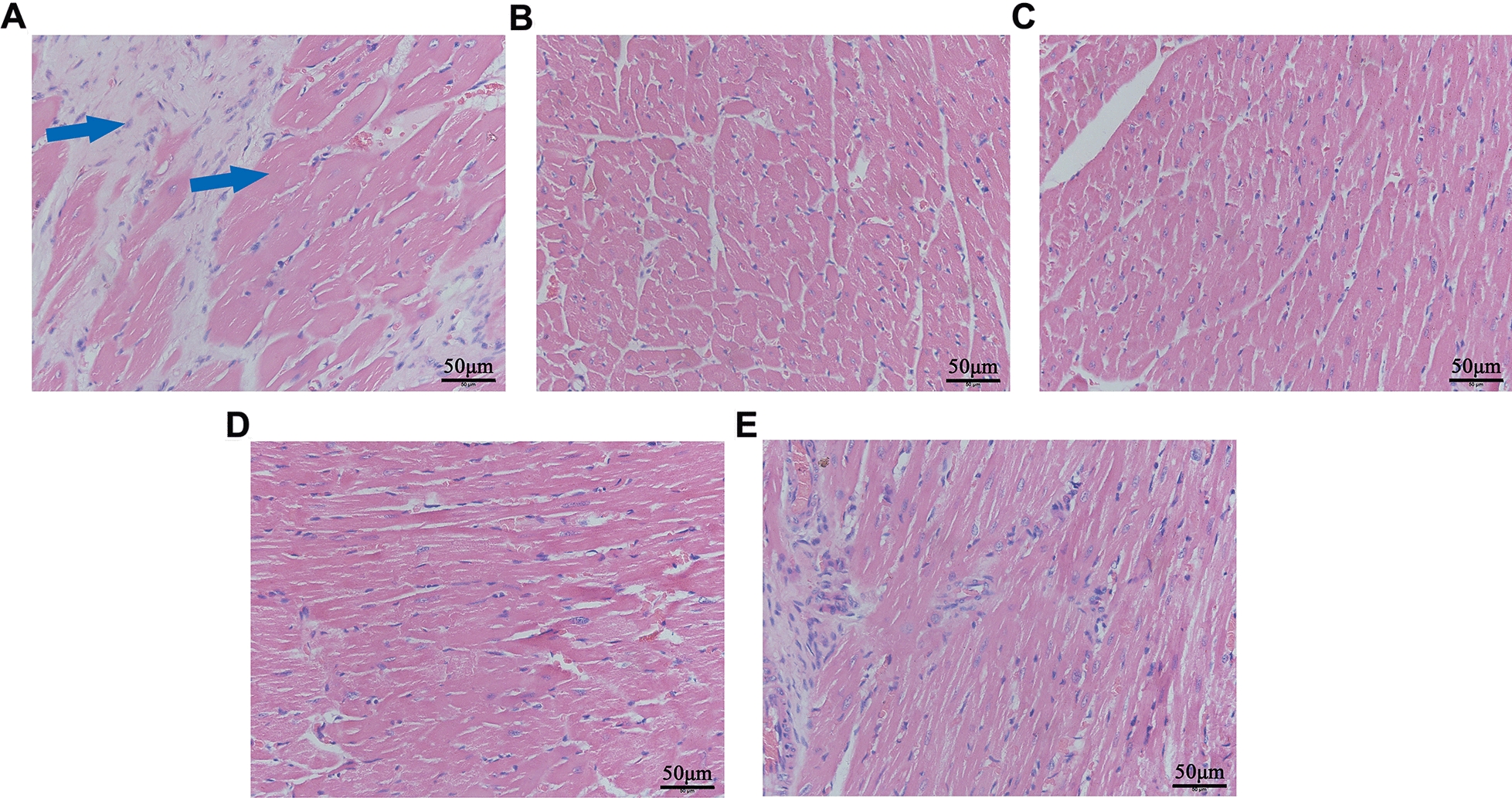
H&E staining results of heart tissue. **A** Model group; **B** Captopril group; **C** High-dose group; **D** Middle-dose group; **E** Low-dose group. Magnification ×400, scale bar = 50 μm. The blue arrows indicate cardiac hypertrophy and fibrosis

As shown in Fig. 4, myocardial samples of the model group developed obvious cardiac hypertrophy and fibrosis, because the cardiomyocytes were swollen and sparse, the area of single cardiomyocyte increased, and the granular content and karyopyknosis in some myocardial cells increased (Fig. 4A). These phenomena were improved in captopril group (Fig. 4B). Compared with the model group, the swelling degree of myocardial cells and area of the single myocardial cell decreased in LFG treatment groups, and three doses of LFG administration reduced the area of myocardial fibrosis to a certain extent (Fig. 4C–E).

### Composite compounds of LFG

**Fig. 5:**
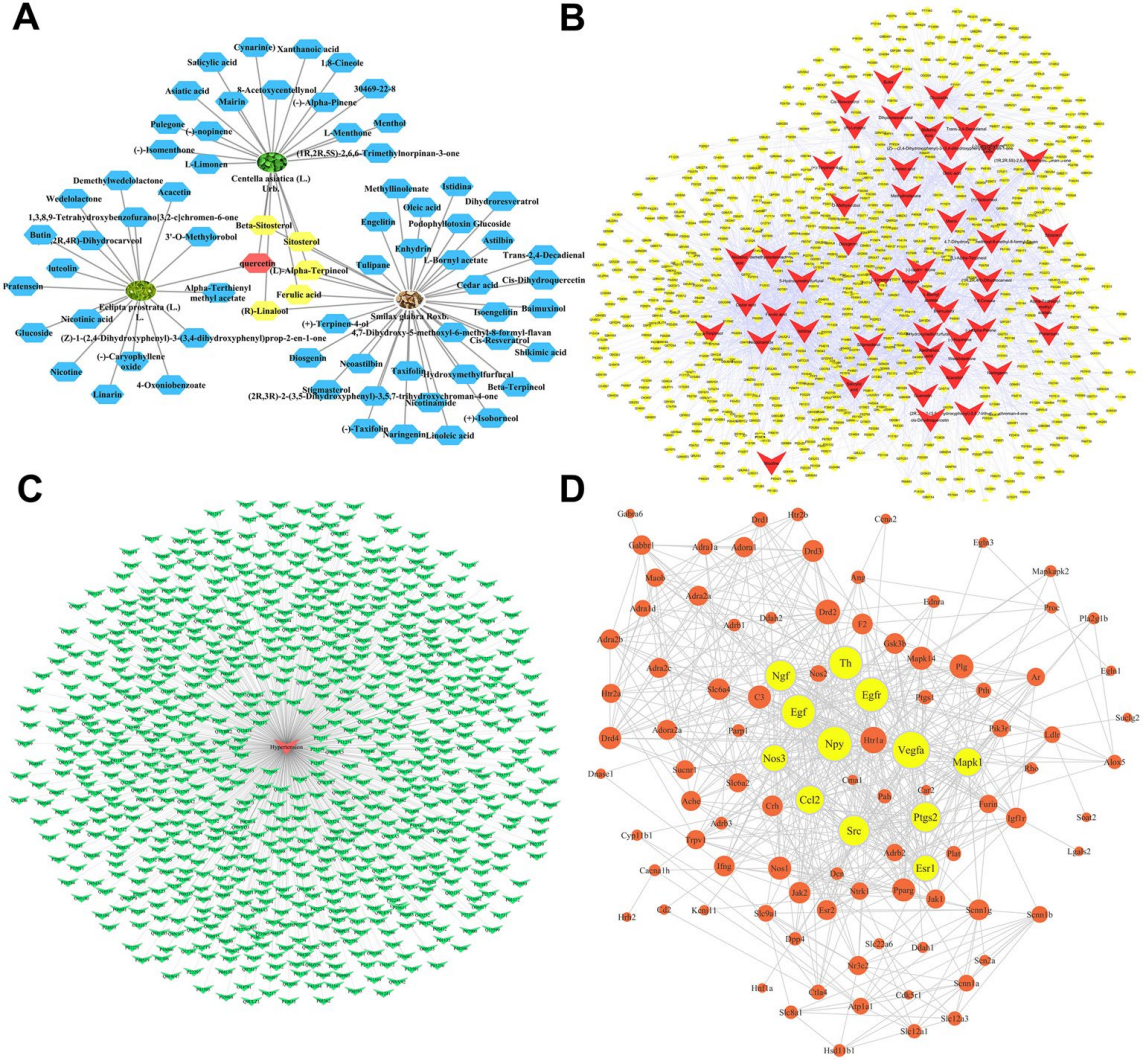
The results of network pharmacology prediction. **A** The herb-compound network. The ellipse nodes represent herbs, and the hexagon nodes are compounds. Among these compounds, yellow parts are the common components of *Centella asiatica* (L.) Urb. and *Smilax glabra* Roxb., and quercetin (red hexagon) is common compounds of 3 herbs. **B** The compound-target network. The V shape nodes represent 53 compounds, and the 765 yellow nodes are targets corresponding to the compounds. **C** The hypertension-target network. In this figure, the red node in the center is hypertension, and the green nodes represent gene targets. **D** The protein-protein interaction network within the common targets. It shows the relationship between 101 common targets. The size of nodes changes from big to small according to degree values, and the 12 yellow nodes are key targets after screening

A total of 70 compounds were collected according to the ADME threshold of OB≥30%, and the herb-compound network was shown in Fig. 5A, which included 73 nodes and 77 edges. The ellipse nodes represented herbs, and the hexagon nodes were compounds. Among these compounds, the yellow parts were common components of *Centella asiatica* (L.) Urb. and *Smilax glabra Roxb.*, and quercetin (red hexagon) was common compounds of 3 herbs.

### Compound-target network

We predicted the putative targets of compounds based on the similarities of drug structures through the ChemMapper database. Since no relevant targets for the 17 herbal compounds were observed, they were removed from 70 herbal compounds. A total of 765 targets were predicted to be hit by the chemical constituents containing in LFG, and the detailed information about the targets was shown in our previous study [22]. The compound-target network was shown in Fig. 5B, the V-shaped nodes represented 53 compounds, and 765 yellow nodes were targets corresponding to the components.

### Hypertension targets and key targets

After collection, 828 hypertension related genes were obtained from T-HOD database (Additional file 1: Table S2). The hypertension-target network was established, as shown in Fig. 5C (the red node in the center was hypertension, and the green nodes represented gene targets). A total of 101 overlapped genes related to candidate compounds and hypertension were kept for PPI network construction using STRING database. The PPI network was visualized by Cytoscape software, as shown in Fig. 5D, in which the size of nodes changes from big to small according to degree values, and the 12 yellow nodes were recognized as the key targets after screening, including NOS3, SRC, prostaglandin G/H synthase 2 (PTGS2), nerve growth factor (NGF), vascular endothelial growth factor A (VEGFA), epidermal growth factor (EGF), epidermal growth factor receptor (EGFR), mitogen-activated protein kinase 1 (ERK2), C-C motif chemokine 2 (CCL2), neuropeptide Y (NPY), estrogen receptor (ESR1), and tyrosine 3-monooxygenase (TH). There were 37 compounds associated with 12 key genes (Table 2).

**Table 2:** The information of 12 key targets between compounds and hypertension targets

Gene	Compounds	Gene	Compounds
VEGFA	Glucoside	MAPK1	Cedar acid
NPY	Ferulic Acid	MAPK1	Ferulic Acid
EGF	Salicylic acid	NOS3	L-Bornyl acetate
EGF	Nicotinic acid	NOS3	p-Hydroxybenzoic acid
EGF	p-Hydroxybenzoic acid	NOS3	Salicylic acid
EGFR	Naringenin	NOS3	Pulegone
TH	(1R,2R,5 S)-2,6,6-trimethylnorpinan-3-one	NOS3	Cedar acid
TH	Ferulic Acid	NOS3	Nicotinic acid
TH	Histidine	NOS3	Histidine
NGF	(-)-alpha-Pinene	NOS3	(R)-linalool
NGF	p-Hydroxybenzoic acid	NOS3	5-Hydroxymethylfurfural
NGF	(-)-nopinene	ESR1	L-Bornyl acetate
SRC	Shikimic Acid	ESR1	(1R,2R,5 S)-2,6,6-trimethylnorpinan- 3-one
SRC	Nicotinic acid	ESR1	Pratensein
SRC	Histidine	ESR1	Alpha-Terthienyl methyl acetate
PTGS2	Naringenin	ESR1	Diosgenin
PTGS2	Histidine	ESR1	(+)-Isoborneol
PTGS2	Demethylwedelolactone	ESR1	Sitosterol
PTGS2	Acacetin	ESR1	Stigmasterol
PTGS2	Ferulic Acid	ESR1	Butin
PTGS2	Xanthanoic acid	ESR1	Baimuxinol
PTGS2	5-Hydroxymethylfurfural	ESR1	(Z)-1-(2,4-dihydroxyphenyl)-3- (3,4-dihydroxyphenyl)prop-2-en-1-one
PTGS2	(L)-alpha-Terpineol	ESR1	(-)-nopinene
PTGS2	(1R,2R,5 S)-2,6,6-trimethylnorpinan-3-one	ESR1	1,8-cineole
PTGS2	Pulegone	ESR1	(-)-alpha-Pinene
PTGS2	MENTHOL	ESR1	Demethylwedelolactone
PTGS2	L-Limonen	ESR1	3’-O-Methylorobol
PTGS2	Salicylic acid	ESR1	Wedelolactone
PTGS2	Nicotinic acid	ESR1	Luteolin
PTGS2	p-Hydroxybenzoic acid	ESR1	Acacetin
PTGS2	L-Bornyl acetate	ESR1	Dihydroresveratrol
PTGS2	Cedar acid	ESR1	Naringenin
CCL2	Ferulic Acid	ESR1	Cis-resveratrol
MAPK1	p-Hydroxybenzoic acid		

### Pathway enrichment of key targets

**Fig. 6:**
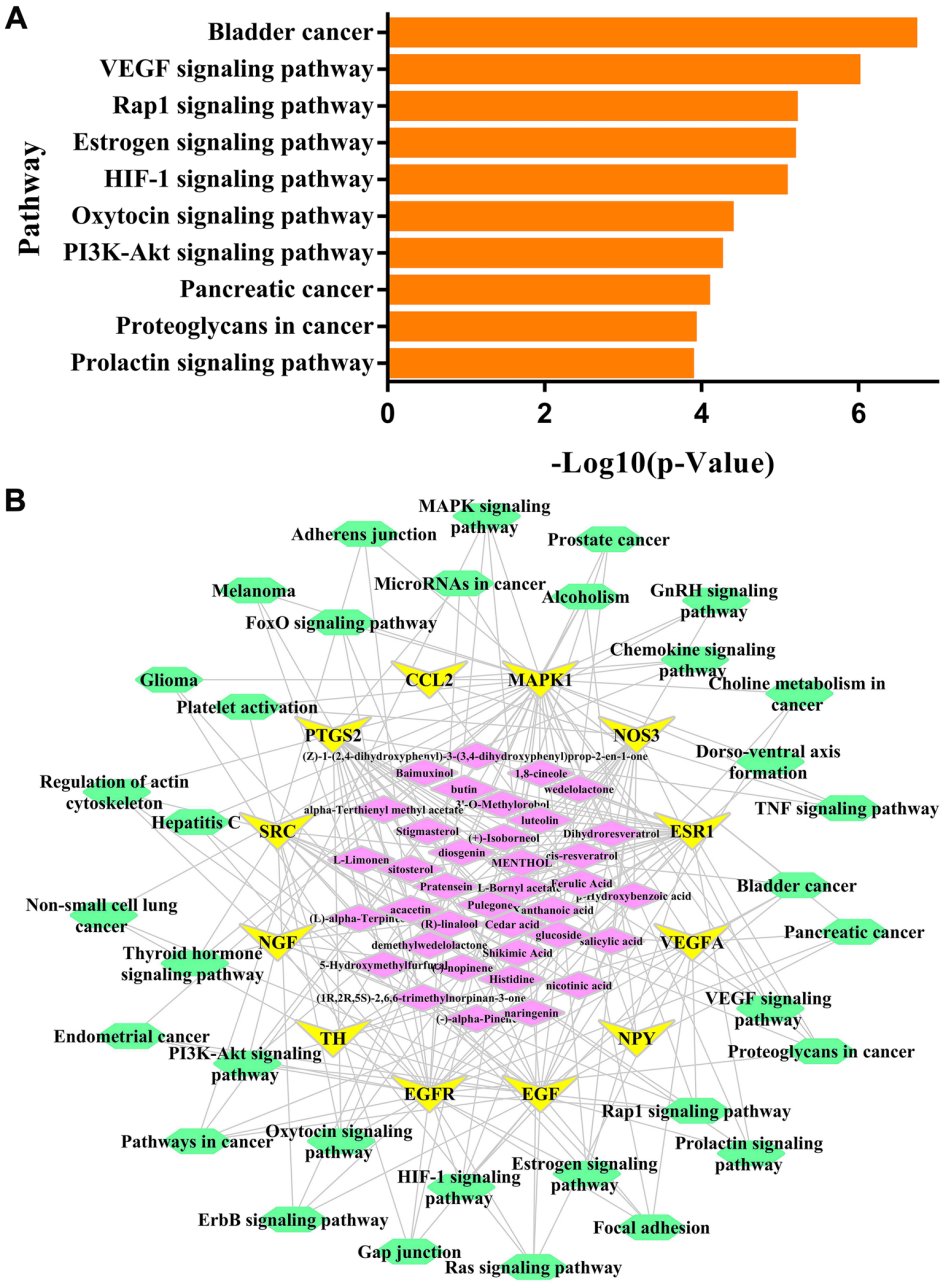
**A** The relationship between TOP10 pathways and their -log10 (P). **B** The compound-target-pathway network. Compounds are indicated by 37 purple nodes; gene targets are shown as 12 yellow nodes; pathways are labeled with 34 green nodes

To clarify the crucial pathways among the key targets in hypertension treatment, KEGG pathway enrichment analysis was performed. The top 10 pathways were shown in Fig. 6A, including Bladder cancer, VEGF signaling pathway, Rap1 signaling pathway, Estrogen signaling pathway, HIF-1 signaling pathway, Oxytocin signaling pathway, PI3K-AKT signaling pathway, Pancreatic cancer, Proteoglycans in cancer, and Prolactin signaling pathway. The compound-target-pathway network was summarized in Fig. 6B, where compounds were indicated by purple nodes; gene targets were shown as yellow nodes; 34 pathways were labeled with green nodes.

### RT-qPCR and western blot verification

**Fig. 7:**
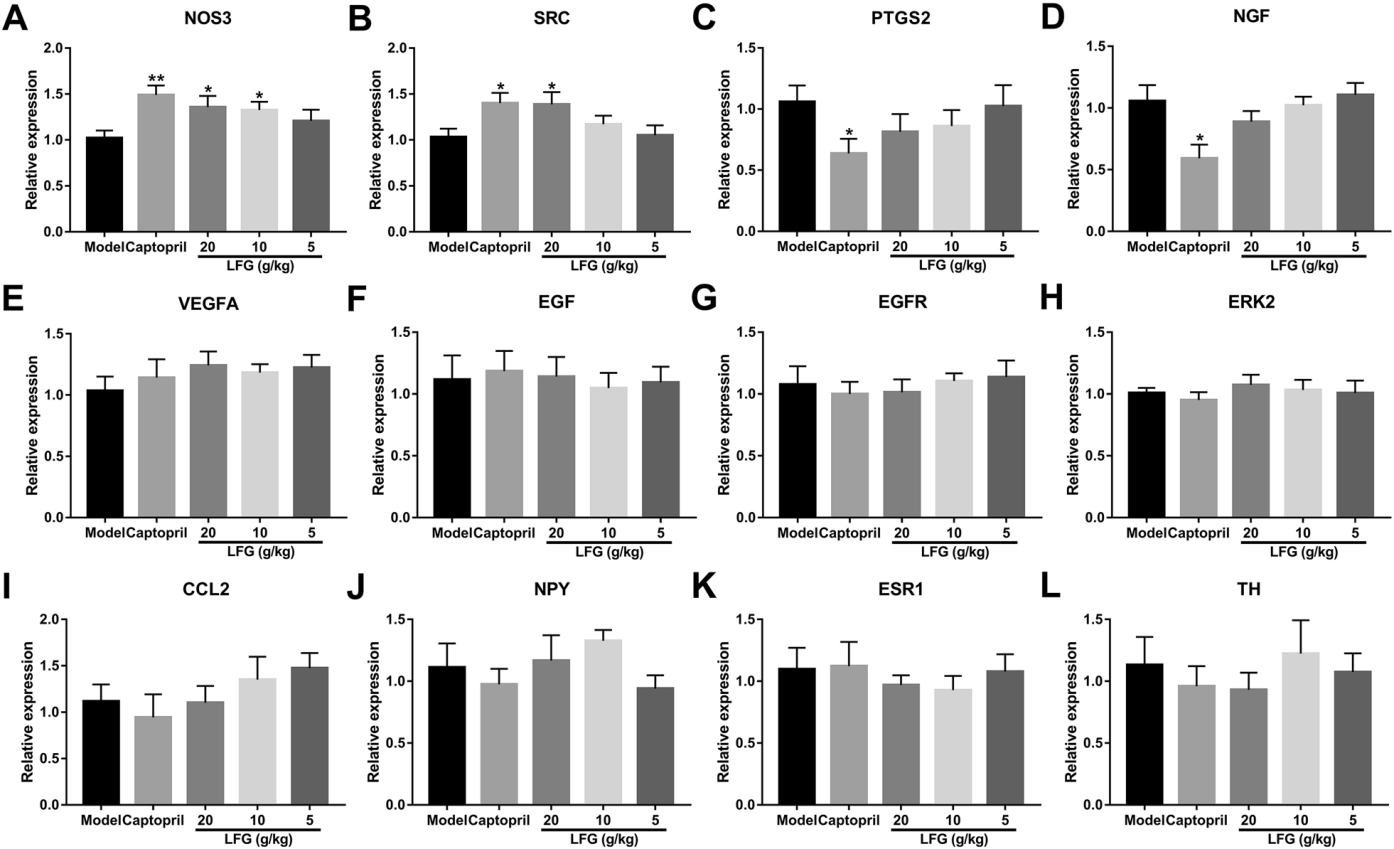
Effect of LFG on mRNA expression levels of key targets. **A** NOS3; **B** SRC; **C** PTGS2; **D** NGF; **E** VEGFA; **F** EGF; **G** EGFR; **H** ERK2; **I** CCL2; **J** NPY; **K** ESR1; **L** TH. Compared with model group, *P < 0.05,**P < 0.01

**Fig. 8:**
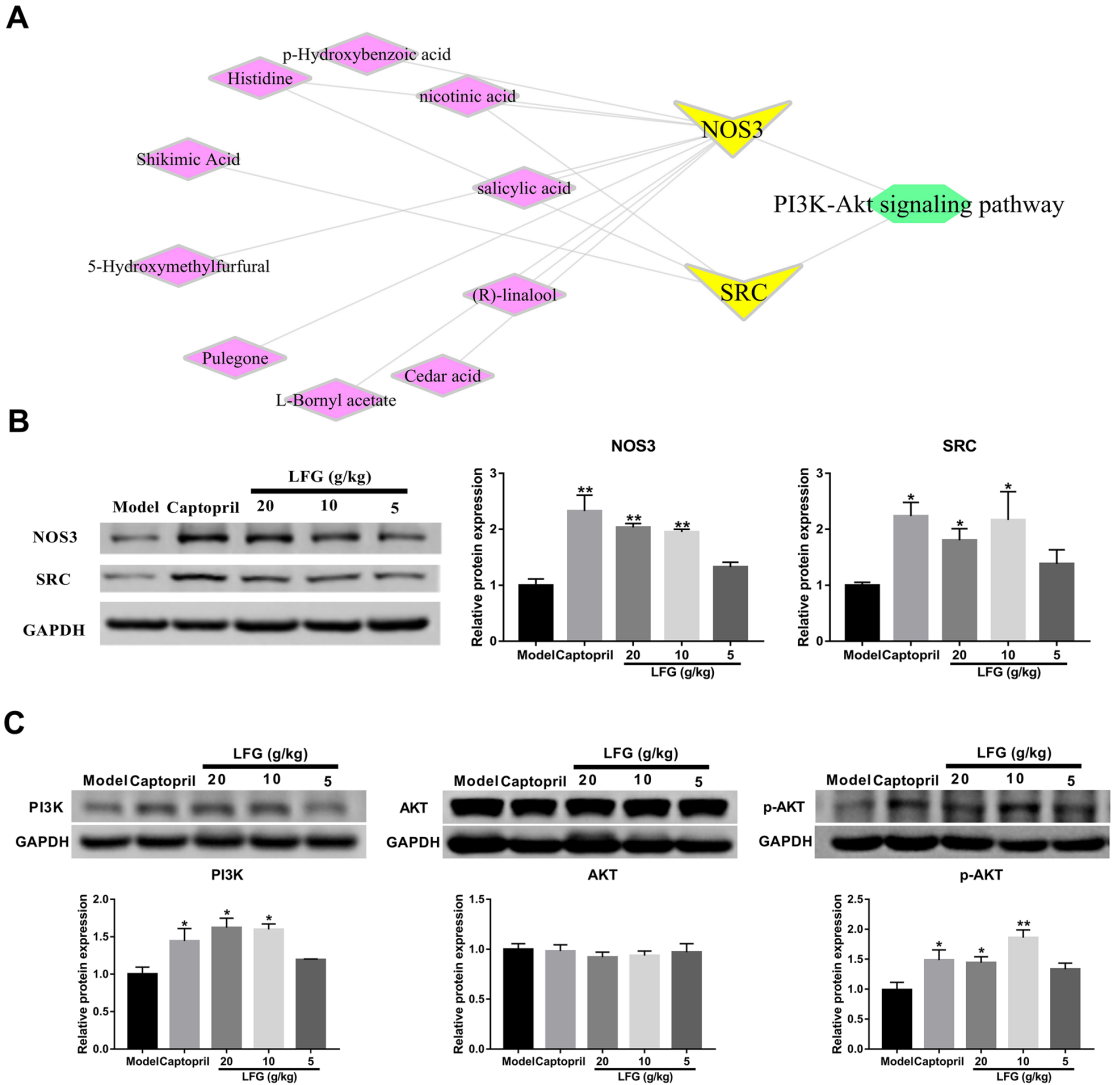
**A** The compound-target-pathway network of significant genes (V nodes). Compounds are indicated by 10 diamond nodes; pathways are shown as hexagon nodes. **B** NOS3 and SRC protein expression levels among different groups. **C** PI3K, AKT and p-AKT protein expression levels among different groups. Compared with model group, * *P* < 0.05, ** *P* < 0.01

To further validate the 12 hub targets predicted from network pharmacology approach, the mRNA expression levels were measured by RT-qPCR. As shown in Fig. 7, the results revealed that the mRNA expression levels of NOS3 and SRC in LFG high-dose group were significantly increased compared with the model group (*P* < 0.01 or 0.05). The mRNA expression level of NOS3 was significantly upregulated in LFG middle-dose group (*P* < 0.05) compared with the model group. For other predicted genes, there was no significant difference between the model group and the LFG treatment groups (*P* > 0.05) (Fig. 7C–L). Therefore, we only focused on NOS3 and SRC in the following study. We further detected the protein levels of NOS3 and SRC in the thoracic aorta tissue. Western blot showed that the protein expression levels of NOS3 and SRC in LFG high- and middle-dose group were significantly increased compared with the model group (*P* < 0.01 or 0.05) (Fig. 8B).

The results of RT-qPCR and western blot showed that LFG could regulate the mRNA and protein expressions of NOS3 and SRC associated with 10 compounds. At the same time, the pathway enrichment analysis of these 2 targets was performed, and the results showed that the antihypertensive effect of LFG might be exerted through the PI3K-AKT signaling pathway. Then we constructed the compound-target-pathway network of significant targets. As shown in Fig. 8A, these compounds were indicated by diamond nodes (p-Hydroxybenzoic acid, Nicotinic acid, Salicylic acid, (R)-Linalool, Cedar acid, L-Bornyl acetate, Pulegone, 5-Hydroxymethylfurfural, Shikimic acid, and Histidine), and the pathways were displayed as hexagon nodes.

To further verify the role of PI3K-AKT signaling pathway in the antihypertensive effect of LFG, the protein expression levels of PI3K, AKT, and p-AKT in the thoracic aorta tissue were measured by western blot analysis. As shown in Fig. 8C, expression levels of PI3K and p-AKT were increased in LFG high- and middle-dose group compared to the model group (*P* < 0.01 or 0.05). There was no significant difference in the level of total AKT expression between the model group and the treatment groups (*P* > 0.05).

### Molecular docking analysis

**Fig. 9:**
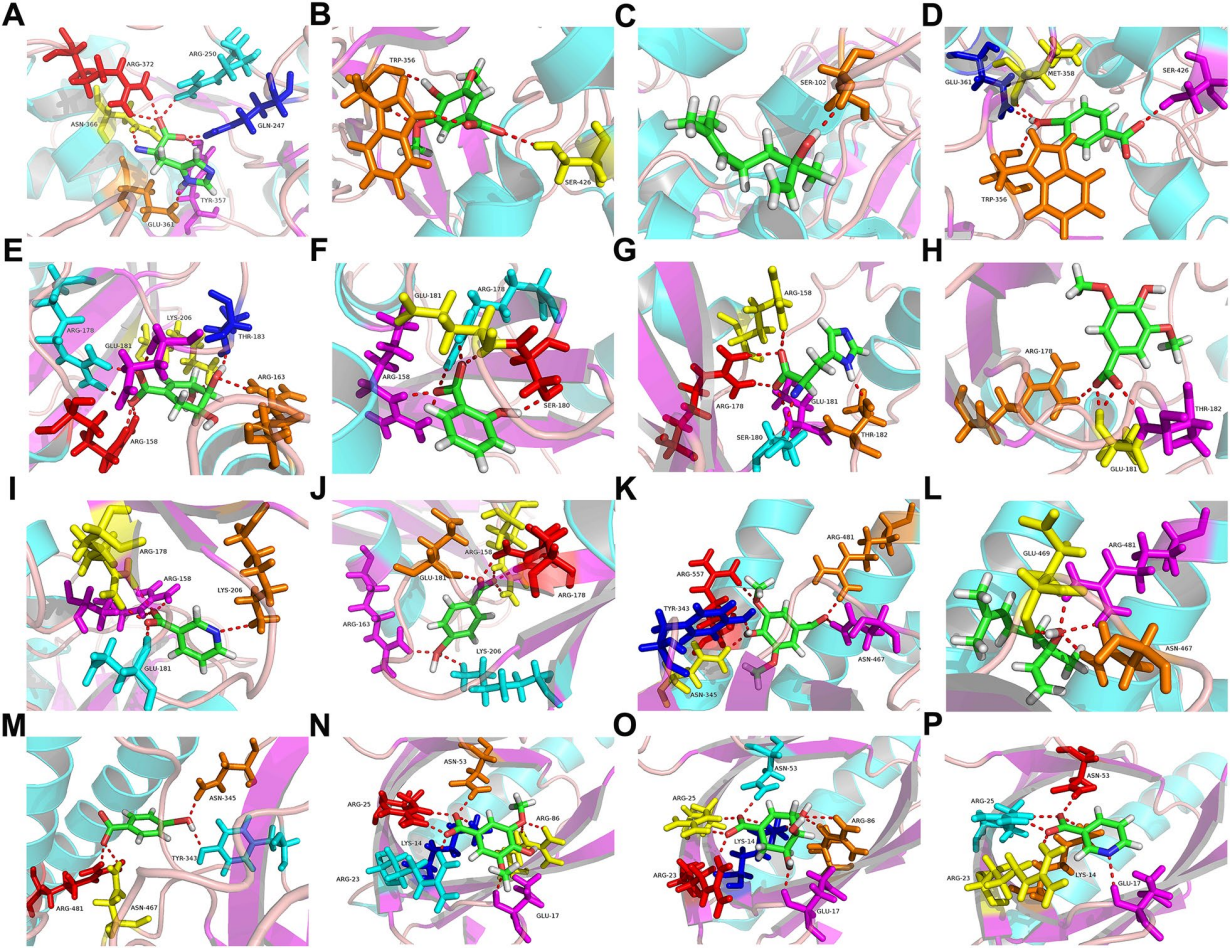
The binding modes of compounds and NOS3: **A** Histidine, **B** Cedar acid, **C** Linalool, **D** p-Hydroxybenzoic acid. The binding modes of compounds and SRC: **E** Shikimic acid, **F** Salicylic acid, **G** Histidine, **H** Cedar acid, **I** Nicotinic acid, **J** p-Hydroxybenzoic acid. The binding modes of compounds and PI3K: **K** Cedar acid, **L** p-Hydroxybenzoic acid, **M** Linalool. The binding modes of compounds and AKT: **N** Cedar acid, **O** Shikimic acid, **P** Nicotinic acid

To elucidate the interaction between targets (NOS3, SRC, PI3K, and AKT) and the 10 potential active compounds, a molecular docking simulation was performed using Sybyl X2.0 to investigate their binding modes. A docking score greater than 6 indicates good protein-ligand binding activity. The docking results of protein-ligand interaction were summarized in Additional file 1: Tables S3–S6. The binding modes of receptors and ligands with good affinity were shown in Fig. 9. In summary, histidine, cedar acid, linalool, and p-hydroxybenzoic acid were successfully docked in NOS3 (Fig. 9A–D). Shikimic acid, salicylic acid, histidine, cedar acid, nicotinic acid, and p-hydroxybenzoic acid were docked in SRC (Fig. 9E–J). Cedar acid, p-hydroxybenzoic acid, and linalool were docked in PI3K (Fig. 9K–M). Cedar acid, shikimic acid, and nicotinic acid were docked in AKT (Fig. 9N–P). Interestingly, cedar acid can be docked in NOS3, SRC, PI3K, and AKT, and p-hydroxybenzoic acid can be docked in NOS3, SRC, and PI3K. The molecular docking results revealed that 7 compounds (Cedar acid, p-hydroxybenzoic acid, histidine, shikimic acid, salicylic acid, linalool, nicotinic acid) exhibited good interaction with NOS3, SRC, PI3K, and AKT.

## LC-MS/MS analysis of LFG

**Fig. 10:**
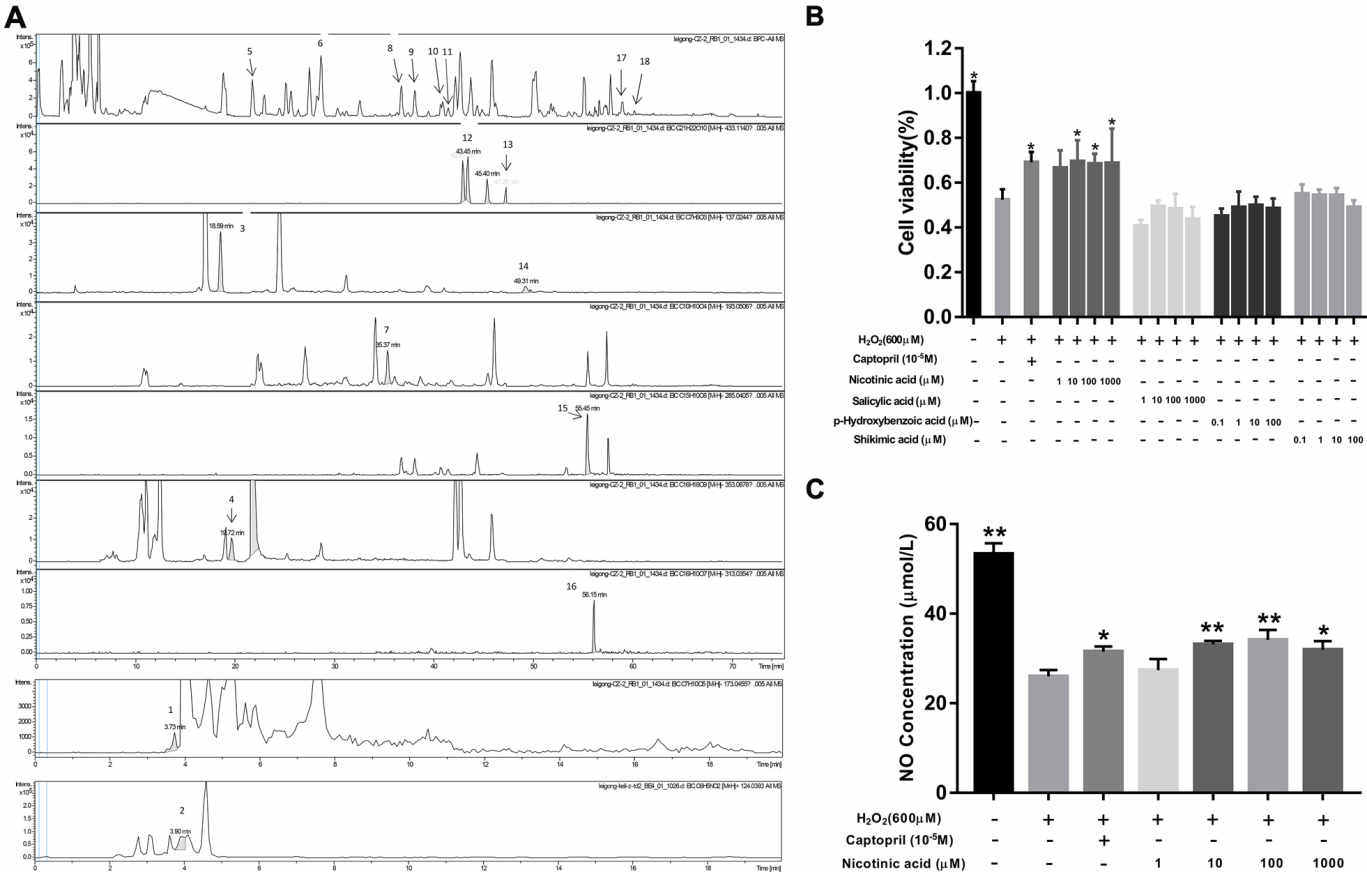
**A** UPLC-QTOF/MS BPI chromatograms of LFG. **B** Protective effects of four compounds against H_2_O_2_ induced oxidative damage in HUVEC. **C** The effect of nicotinic acid on NO release in HUVEC. Compared with H_2_O_2_-induced group, *P < 0.05,**P < 0.01

In order to verify whether the 7 components predicted by molecular docking exist in LFG, LC-MS/MS was p';/erformed to analyze the chemical constituents of LFG. The representative base peak intensity (BPI) chromatogram in negative ion mode was shown in Fig. 10 L.A. After comparing each component in LFG with the reference standard (Additional file 2: Figure S1–S18), 18 compounds were detected, and the detail information of these compounds was shown in Table 3. Among these compounds, four compounds (p-hydroxybenzoic acid, shikimic acid, salicylic acid, nicotinic acid) were confirmed to exist in LFG and they docked well with NOS3, SRC, PI3K, and AKT. In addition, we determined the contents of above 4 compounds in LFG, and the results showed the contents of p-hydroxybenzoic acid, shikimic acid, salicylic acid and nicotinic acid in LFG were 100, 1360, 36 and 36.8 µg/g respectively.

**Table 3:** Identify of the compounds of LFG by UPLC- Q-TOF/MS analysis in negative ion mode

NO.	R-T	m/z	MS/MS	Elemental composition	Ion form	ppm	Compounds
1	3.73	173.0458	93.0354	C_7_H_10_O_5_	[M-H]^−^	− 1.3	Shikimic acid
2	3.90	124.0394	78.0334/80.0490	C_6_H_5_NO_2_	[M-H]^+^	− 0.7	Nicotinic acid
3	18.59	137.0244	93.0354	C_7_H_6_O_3_	[M-H]^−^	0.2	p-Hydroxybenzoic acid
4	19.72	353.0882	191.0565	C_16_H_18_O_9_	[M-H]^−^	− 1.0	Chlorogenic acid
5	21.78	353.0881	191.0563/173.0456	C_16_H_18_O_9_	[M-H]^−^	− 0.9	4-Dicaffeoylquinic acid
6	28.69	515.1196	191.0563/179.0351	C_25_H_24_O_12_	[M-H]^−^	− 0.1	Isochlorogenic acid C
7	35.37	193.0509	106.0438/134.0376	C_10_H_10_O_4_	[M-H]^−^	− 1.4	Ferulic acid
8	36.84	449.1089	151.0036/285.0405	C_21_H_22_O_11_	[M-H]^−^	0.1	Neoastilbin
9	38.09	449.1099	151.0040/285.0413	C_21_H_22_O_11_	[M-H]^−^	− 2.3	Astilbin
10	40.67	449.1095	151.0037/285.0410	C_21_H_22_O_11_	[M-H]^−^	− 1.3	Neoisoastilbin
11	41.50	449.1094	151.0037/285.0408	C_21_H_22_O_11_	[M-H]^−^	− 0.9	Isoastilbin
12	43.45	433.1147	180.0068/269.0463	C_21_H_22_O_10_	[M-H]^−^	− 1.6	Engeletin
13	47.33	433.1137	180.0059/269.0448	C_21_H_22_O_10_	[M-H]^−^	− 0.4	Isoengelitin
14	49.31	137.0245	93.0354	C_7_H_6_O_3_	[M-H]^−^	− 0.5	Salicylic acid
15	55.45	285.0409	133.0297/151.0033	C_15_H_10_O_6_	[M-H]^−^	− 1.7	Luteolin
16	56.15	313.0368	298.0126	C_16_H_10_O_7_	[M-H]^−^	− 4.4	Wedelolactone
17	59.00	503.3381	389.2861/437.3066	C_30_H_48_O_6_	[M-H]^−^	− 0.6	Madecassic-acid
18	60.20	487.3431	379.3034/409.3156	C_30_H_48_O_5_	[M-H]^−^	− 0.4	Asiatic acid

### Protective effect of compounds

To further verify the antihypertensive effect of p-hydroxybenzoic acid, shikimic acid, salicylic acid and nicotinic acid in LFG. We detected the protective effect of 4 compounds on HUVEC *in vitro*. As shown in Fig. 10B, the cell viability in the H_2_O_2_-induced group was decreased significantly, whereas the cell viability in the groups treated with nicotinic acid (10 µM, 100 µM, 1000 µM) were increased (*P* < 0.05). Compared with H_2_O_2_-induced group, the NO level in cell supernatant of nicotinic acid (10 µM, 100 µM, 1000 µM) groups were increased significantly (Fig. 10C) (*P* < 0.01 or 0.05). These results indicated that nicotinic acid exhibited protective effect on HUVEC and might be the potential active compound for LFG antihypertensive effect.

## Discussion

Hypertension is a major risk factor for cardiovascular and cerebrovascular diseases as current complications, such as stroke, heart failure, myocardial infarction and renal failure [34]. Blood pressure can be controlled by changing diet and lifestyle, but drug treatment is used when these methods are ineffective. Drugs used to treat hypertension include calcium channel blockers, β-blockers, angiotensin converting enzyme inhibitors (ACEI) and angiotensin II receptor blockers. However, various side effects of these drugs have been reported [35, 36]. Long-established Chinese herbal formulas can stabilize blood pressure, and improve the quality of life, minimize the risk factors associated with high blood pressure, and prevent organ damage to improve patient survival [37]. In this study, we used a SHR model to assess the effects of LFG on attenuating blood pressure. Our data showed that LFG could significantly reduce blood pressure and increase serum NO content in SHRs compared with the model group. LFG ameliorated pathological changes such as cardiac hypertrophy and interstitial inflammation.

Although the animal experimental results showed that LFG could exert an antihypertensive effect in SHR, the active compounds and underlying molecular mechanisms are not clear. In the present study, a network pharmacology approach was applied to identify the active compounds, corresponding targets, and pharmacological mechanisms of LFG antihypertensive effect. Based on the results of public databases searching, 53 candidate compounds were collected from LFG, which were linked to 765 potential targets. A total of 828 hypertension associated targets were retrieved. The potential targets of antihypertensive effect were found by comparing LFG genes with hypertension genes, and 12 key targets were obtained after screening. After pathway enrichment of 12 key targets, 37 components and 34 pathways were found, among which the top 10 pathways were Bladder cancer, VEGF signaling pathway, Rap1 signaling pathway, Estrogen signaling pathway, HIF-1 signaling pathway, Oxytocin signaling pathway, PI3K-AKT signaling pathway, Pancreatic cancer, Proteoglycans in cancer, and Prolactin signaling pathway.

Twelve key genes were validated by RT-qPCR and western blot, and the results showed that LFG could upregulate the mRNA and protein expressions of NOS3 and SRC in thoracic aorta. NOS3 is mainly expressed in endothelial cells. Due to its vasodilator, antioxidant and antiproliferative properties, NO produced by NOS3 has become the main vascular protective agent [38]. It was reported that demethylasterriquinone b1 induced AKT activation may stimulate the expression and activity of NOS3 and inhibit the expression of p22phox subunit of NADPH oxidase from reducing oxidative stress and subsequently improve vascular endothelial dysfunction [39]. SRC tyrosine protein kinase is a non-receptor protein tyrosine kinase. It plays an important role in regulating cell differentiation, proliferation, and transformation, and also plays an important biological role in diseases such as hypertension and tumor [40, 41]. It was reported that SRC was involved in the production of NO and prostaglini2 (PGI2) induced by VEGF, suggesting that SRC was involved in reducing vascular tension, relaxing blood vessels, and lowering blood pressure of VEGF in the vascular endothelium [42].

VEGF is a key protein regulator of angiogenesis under physiological and pathological conditions, and it may be important for vascular protection mechanism in hypertension [43]. Epidermal growth factor (EGF) is produced in different tissues and exerts stimulatory effects on cell growth, proliferation and survival. Vascular remodeling is caused by increased smooth muscle proliferation and may be due to an increase in the responsiveness of vascular cells to EGF [44]. EGF binds to its receptor (EGFR), which was identified as a plasma membrane protein tyrosine kinase in 1980 [45]. It was suggested that EGFR signaling is enhanced in various animal models of hypertension [46]. Prostaglandin G/H synthase 2 (PTGS2) also named Cyclooxygenase-2 (COX-2) is important in pathological processes such as inflammation. Hypertension is associated with augmented vascular COX-2 expression and COX-2-derived prostanoids [47]. Nerve growth factor (NGF) belongs to the neurotrophin family and plays an essential role in neuronal development, survival, and differentiation. Elevated NGF may be involved in developing hypertension in SHR [48]. Mitogen-activated protein kinase 1(MAPK1) also named Extracellular signal-regulated kinase 2 (ERK2) plays an essential role in the MAPK/ERK cascade. Depending on the cellular context, the MAPK/ERK cascade mediates diverse biological functions such as cell growth, adhesion, survival and differentiation through the regulation of transcription, translation, cytoskeletal rearrangements. A study reported that Bu-Shen-Jiang-Ya decoction (BSJYD) treatment decreased blood pressure and heart rate efficiently and reversed ventricular remodeling of SHR, and the mechanisms were possibly associated with the suppressive effect of BSJYD on the EKR signaling pathway [49]. C–C motif chemokine 2 (CCL2) also named Monocyte chemotactic protein 1(MCP-1) is one of the chemotactic factors produced by damaged endothelial cells during the development of atherosclerosis. MCP-1 through the activation of CCR2 can induce migration and attachment of monocytes/macrophages, lymphocytes, endothelial cells and vascular smooth muscle cells [50]. Tyrosine hydroxylase (TH), a key enzyme in the catecholamine synthetic pathway, is increased in the adrenal medulla of SHR [51]. Neuropeptide Y (NPY) is a 36-amino acid peptide neurotransmitter, released from postganglionic sympathetic perivascular neurons [52]. Alternations of NPY release may play a role in the pathophysiology of at least some forms of hypertension [53]. Estrogen receptor 1 (ESR1) is a nuclear hormone receptor. The steroid hormones and their receptors are involved in regulating eukaryotic gene expression and affect cellular proliferation and differentiation in target tissues. It was demonstrated that ESR1 plays an important role in pulmonary arterial hypertension [54]. Taken together, the above 12 key targets were closely related to hypertension, among which the levels of NOS3 and SRC were restored by LFG.

**Fig. 11:**
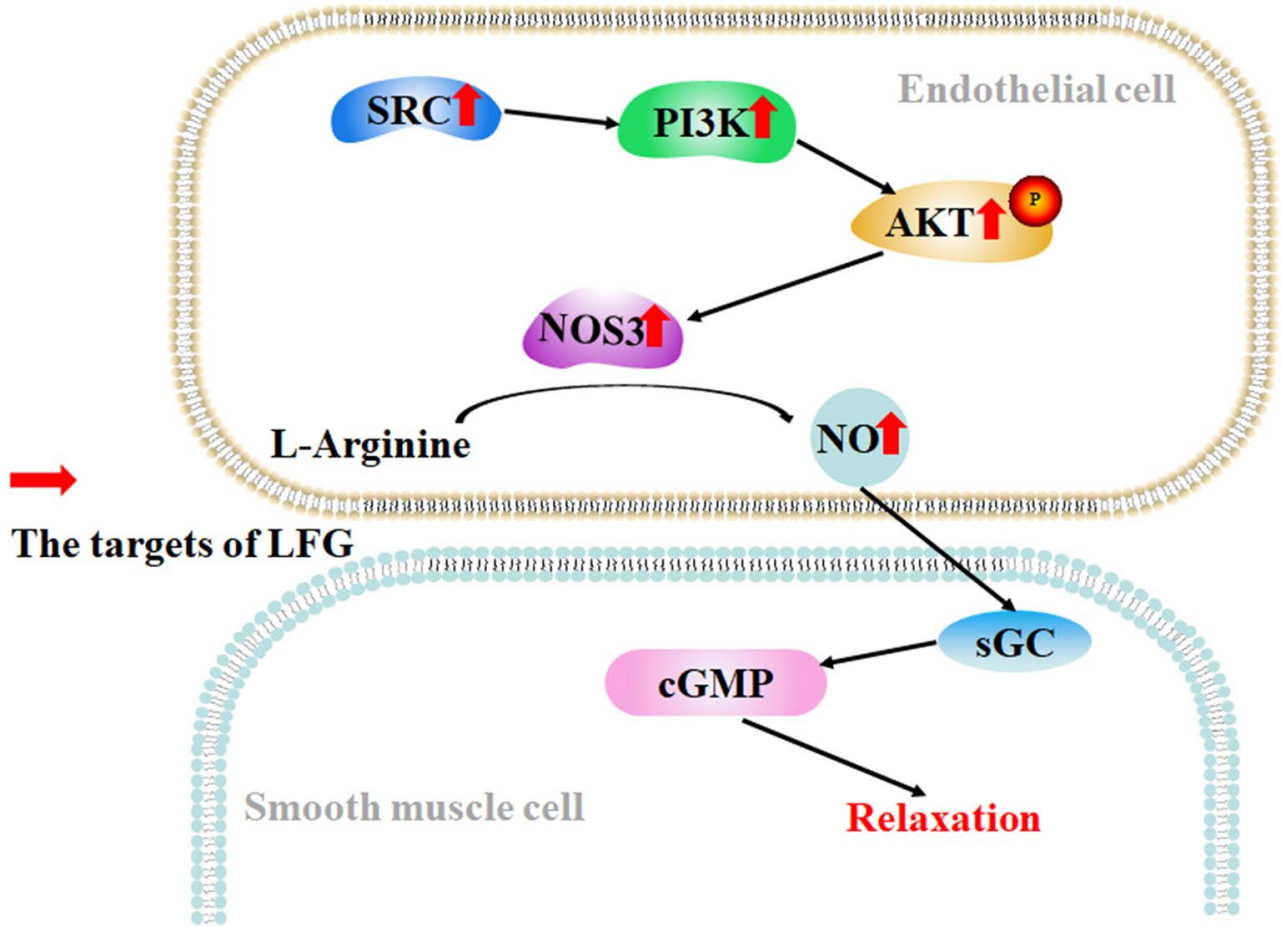
LFG antihypertensive mechanism

Pathway enrichment results showed that the PI3K-AKT signaling pathway was closely related to the expression of NOS3 and SRC. To verify this result, western blot was used to detect the key proteins (PI3K, AKT, and p-AKT) of the PI3K-AKT signaling pathway. The results showed that LFG significantly increased the protein expression levels of PI3K and p-AKT in SHRs, suggesting that LFG may play a role through the PI3K-AKT signaling pathway. The PI3K-AKT signaling pathway is activated by various types of cellular stimuli or toxic insults and regulates fundamental cellular functions such as transcription, translation, proliferation, growth, and survival. PI3K catalyzes the production of phosphatidylinositol-3,4,5-triphosphate (PIP3) on the cell membrane. In turn, PIP3 serves as a second messenger to help activate AKT. Once activated, AKT can control key cellular processes by phosphorylating substrates in apoptosis, protein synthesis, metabolism, and cell cycle. It has been reported that the AKT activity of blood vessels characterized by Ser473 phosphorylation is downregulated in a number of hypertensive rat models, including SHRs [55]. Importantly, phosphorylation of AKT at Ser473 contributes to NOS3 activation [56]. SRC regulates the activity of PI3K-AKT signaling, and contributes to improving endothelial dysfunction [57]. Taken together, the mechanism of LFG exerting antihypertensive effect was showed in Fig. 11. LFG can lower the blood pressure of SHRs, which might be attributed to upregulating SRC expression level, activating the PI3K-AKT-NOS3 signaling pathway to further increase the serum NO level for promoting vasodilation.

Molecular docking studies further supported that p-hydroxybenzoic acid, cedar acid, shikimic acid, salicylic acid, nicotinic acid, linalool, and histidine can bind well with NOS3, SRC, PI3K, and AKT. These compounds were the potential active ingredients for LFG antihypertensive effect. p-Hydroxybenzoic acid can be isolated from a variety of plants, such as oil palm, Vitex negundo and virgin olive oil [58–60]. It was reported that p-hydroxybenzoic acid possessed a variety of biological properties, including anti-inflammatory, antioxidant cardio protective and vasodilatory effects [61]. p-Hydroxybenzoic acid causes a decrease in blood pressure and dilatation of the aortic rings. The biological principles were to act directly on the vascular smooth muscle to cause vasodilatation, and indirectly by stimulating the release of NO from the vascular endothelium to promote the vasodilator activity of the compound [62]. Cedar acid is one of the phenolic compounds widely found in olives, dates, spices, pumpkin, grapes, acai palm and other plants [63]. In addition, it exhibits multi-pharmacological properties such as antihypertensive, antioxidant, anti-inflammatory, and hepatoprotective activities [64, 65]. Cedar acid showed antihypertensive activity in hypertensive rats induced by N-nitro-L-arginine methyl ester (L-NAME). It was evidenced that Cedar acid treatment might reduce the blood pressure, lipid peroxides, and increase NO availability and antioxidant levels in blood samples of rats [66]. Shikimic acid originally extracted from Chinese star anise, exerts diverse pharmacological actions such as analgesic, anti-inflammatory, and antioxidant effects [67–69]. Previous studies indicated that Shikimic acid inhibited inflammatory events including the production of NO and the expression of pro-inflammatory cytokines [70]. Chemically, Salicylic acid is 2-hydroxybenzoic acid or orthohydrobenzoic acid. Sources of Salicylic acid include willow bark, sweet birch, and wintergreen leaves, and it can also be synthesized artificially [71]. Salicylic acid, the Xinjiang red raspberry fruit extract, moderately reduced SHR blood pressure, the effect may be related to increasing serum NO, SOD activity and T-AOC levels, enhanced NOS3 mRNA expression [72]. Niacin, also known as vitamin B3, illustrates the many functions of water-soluble vitamins. Niacin has been widely used in the treatment of dyslipidemia and atherosclerotic coronary heart disease [73]. In addition to its lipid-lowering effect, Niacin also has powerful antioxidant and anti-inflammatory properties [74]. Cho etc. showed that long-term Niacin administration improves hypertension, proteinuria and attenuates the accumulation of lipids in the remnant kidneys of animals with CRF induced by subtotal nephrectomy [75]. Linalool is a monoterpene and is generally consideredthe main component of essential oils obtained from aromatic plant species [76]. Linalool showed a wide range of biological activities including anti-inflammatory, anti-cancer, anti-hyperlipidemic, anti-microbial effects [77]. Data showed that the use of Linalool for sub-chronic treatment could reduce the development of hypertension in SHRs, reduce cardiac hypertrophy, increase IL-10 anti-inflammatory cytokine, and improve vasodilatory function and decreasing vasoconstriction [78]. Histidine is an essential amino acid in humans and other mammals which exhibits antioxidant and anti-inflammatory properties [79]. In dietary intervention and evaluation experiment, the relationship between the intake of specific amino acids and blood pressure levels was analyzed, and the results demonstrated that intakes of Methionine and Alanine were positively correlated with higher blood pressure, while intakes of Threonine and Histidine had inverse associations [80]. It has been proved that L-histidine has an antihypertensive effect after oral administration in SHRs. These effects may be mediated by central histamine H3 receptor, which seems to be related to an increase in NO in the rostral ventrolateral medulla [81].

After comparing the components with the reference standard and LFG by UPLC-MS/MS, it can be determined that p-hydroxybenzoic acid, shikimic acid, salicylic acid, and nicotinic acid were presented in LFG. In addition, the contents of above 4 compounds in LFG were relatively high. Therefore, it is possible that p-hydroxybenzoic acid, shikimic acid, salicylic acid, and nicotinic acid contribute to the therapeutic effect of LFG. Cell viability assay *in vitro* suggested that nicotinic acid have substantial protective effects against the reduction in endothelial cell viability caused by H_2_O_2_ treatment, and it could promote the release of NO from HUVEC. In conclusion, nicotinic acid might be the basis of the antihypertensive effect of LFG.

## Conclusions

LFG can lower the blood pressure of SHRs, which might be attributed to the upregulation of SRC and activation of the PI3K-AKT-NOS3 signaling pathway, which further increases the serum NO level, thereby promoting vasodilation. Nicotinic acid might be the underlying active compound for LFG antihypertensive effect.

## Supplementary Information

**Additional file 1: Table S1.** The contents of 14 compounds obtained from literatures. **Table S2.** The 828 significant genes associated with hypertension. **Table S3.** Docking results of 10 active ingredients and NOS3. **Table S4.** Docking results of 10 active ingredients and SRC. **Table S5.** Docking results of 10 active ingredients and PI3K. **Table S6.** Docking results of 10 active ingredients and AKT.

**Additional file 2: Figures S1-S18.** The mass spectrum comparison of components in LFG with the reference standard.

## Data Availability

The datasets used and/or analyzed during the current study are available from the corresponding author on reasonable request.

## Abbreviations

AKT: Protein kinase B
ANG-II: Angiotensin II
BATMAN-TCM: Bioinformatics Analysis Tool for Molecular Mechanism of Traditional Chinese Medicine
BCA: Bicinchoninic acid
BSA: Bovine Serum Albumin
CCL2: C-C motif chemokine 2
COX-2: Cyclooxygenase-2
DAVID: Database for Annotation, Visualization and Integrated Discovery
EGF: Epidermal growth factor
EGFR: Epidermal growth factor receptor
ERK: Mitogen-activated protein kinase
ESR1: Estrogen receptor 1
ET-1: Endothelin 1
H&E staining: Hematoxylin-eosin staining
H_2_O_2_: hydrogen peroxide
HUVEC: human umbilical vein endothelial cells
KEGG: Kyoto Encyclopedia of Genes and Genomes
LFG: Lei-gong-gen formula granule
MAPK1: Mitogen-activated protein kinase
MCP-1: Monocyte chemotactic protein 1
NGF: Nerve growth factor
NO: Nitric oxide
NOS3: Endothelial nitric oxide synthase
NPY: neuropeptide Y
OB: Oral bioavailability
PDB: Protein Data Bank
PI3K: Phosphatidylinositol 3 kinase
PTGS2: Prostaglandin G/H synthase 2
SHR: Spontaneously hypertensive rats
SRC: Proto-oncogene tyrosine-protein kinase SRC
STRING: Search Tool for the Retrieval of Interacting Genes/Proteins
TCMID: Traditional Chinese Medicines Integrated Database
TCMSP: Traditional Chinese Medicine Systems Pharmacology Database and Analysis Platform
TH: Tyrosine hydroxylase
T-HOD: Text-mined Hypertension, Obesity and Diabetes candidate gene database
UPLC-QTOF/MS: Ultra performance liquid chromatography/quadrupole time-of-flight mass spectrometry
VEGFA: Vascular endothelial growth factor A
