# Supplementary material for: A network pharmacology-based approach to explore the active ingredients and molecular mechanism of Lei-gong-gen formula granule on a spontaneously hypertensive rat model
Source: Chin Med. 2021 Oct 9;16:99. doi: 10.1186/s13020-021-00507-1 (PMC8501634; doi:10.1186/s13020-021-00507-1)
Supplement: Supplementary file 1 — Additional file 1: Table S1. The contents of 14 compounds obtained from literatures. Table S2. The 828 significant genes associated with hypertension. Table S3. Docking results of 10 active ingredients and NOS3. Table S4. Docking results of 10 active ingredients and SRC. Table S5. Docking results of 10 active ingredients and PI3K. Table S6. Docking results of 10 active ingredients and AKT. [file 13020_2021_507_MOESM1_ESM.docx]

**Table S1: The contents of 14 compounds obtained from literatures**

| **Number** | **Ingredients** | **Herbal medicine** | **Yield (mg/g)** | **Reference** |
| --- | --- | --- | --- | --- |
| 1 | Diosgenin | *Smilax glabra Roxb.* | 0.024-0.035 | He P., Ren G.C., Wei X., Dong L.S., 2010. Determination of diosgenin in *Smilax glabra* Roxb*.*from Guizhou different habitat. Studies of Trace Elements and Health 4, 47-48. |
| 2 | (L)-Alpha-Terpineol | *Centella asiatica (L.) Urb.* | 0.0012 | Joshi V.P., Kumar N., Singh B., Chamoli R.P., 2007. Chemical composition of the essential oil of *Centella asiatica* (L.) Urb. from western Himalaya. Natural Product Communication 5, 587-510. |
| 3 | Quercetin | *Centella asiatica (L.) Urb.* | 1.28-3.17 | Yan K., Xu Y.M., Xu J.P., Duan T.W., Li D.Q., 2017. Determination of seven active components in *Centella asiatica (L.) Urb.*by UPLC-MS/MS. The Chinese Journal of Clinical Pharmacology 19, 1949-1953. |
| 4 | (-)-Alpha-Pinene | *Centella asiatica (L.) Urb.* | 0.02094 | Oyedeji, O.A., Afolayan, A.J. 2005. Chemical composition and antibacterial activity of the essential oil of Centella asiatica. Growing in South Africa. Pharmaceutical biology 3, 249-252. |
| 5 | (-)-Nopinene | *Centella asiatica (L.) Urb.* | 0.003 | Joshi V.P., Kumar N., Singh B., Chamoli R.P., 2007. Chemical composition of the essential oil of *Centella asiatica* (L.) Urb. from western Himalaya. Natural Product Communication 5, 587-510. |
| 6 | Asiatic acid | *Centella asiatica (L.) Urb.* | 2.43-3.46 | Yan K., Xu Y.M., Xu J.P., Duan T.W., Li D.Q., 2017. Determination of seven active components in *Centella asiatica (L.) Urb.*by UPLC-MS/MS. The Chinese Journal of Clinical Pharmacology 19, 1949-1953. |
| 7 | Neoastilbin | *Smilax glabra Roxb.* | 0.182-0.317 | Yuan M.H., 2018. Effect of sulfur fumigation on the quality of *Smilax glabra Roxb.* (Master's thesis), Southwest Minzu University, 33. |
| 8 | Linalool | *Centella asiatica (L.) Urb.* | 0.0024 | Joshi V.P., Kumar N., Singh B., Chamoli R.P., 2007. Chemical composition of the essential oil of *Centella asiatica* (L.) URB. from western Himalaya. Natural Product Communication 5, 587-510. |
| 9 | 1,8-Cineole | *Centella asiatica (L.) Urb.* | 0.0024 | Joshi V.P., Kumar N., Singh B., Chamoli R.P., 2007. Chemical composition of the essential oil of *Centella asiatica* (L.) Urb. from western Himalaya. Natural Product Communication 5, 587-510. |
| 10 | Ferulic acid | *Smilax glabra Roxb.* | 0.05964 | Sun J.Z., 2015. Determination of colchicine, ferulic acid and resveratrol in *Smilax glabra Roxb.* by HPLC. Shaanxi Journal of Traditional Chinese Medicine 1, 103-104. |
| 11 | Beta-Sitosterol | *Smilax glabra Roxb.* | 0.765-0.813 | Jiang C., Huang Y.F., Zhang L., Zhuo S., Lin X., 2019. Effects of three different processing methods on the content of β-sitosterol in rhizoma heterosmilacis japonicae. China Medicine and Pharmacy 23, 34-37. |
| 12 | Astilbin | *Smilax glabra Roxb.* | 1.86-2.10 | Yuan M.H., 2018. Effect of sulfur fumigation on the quality of *Smilax glabra Roxb.* (Master's thesis), Southwest Minzu University, 33. |
| 13 | Engelitin | *Smilax glabra Roxb.* | 0.0129-0.0188 | Yuan M.H., 2018. Effect of sulfur fumigation on the quality of *Smilax glabra Roxb.* (Master's thesis), Southwest Minzu University, 33. |
| 14 | Luteolin | *Eclipta prostrata (L.) L.* | 0.17-0.48 | Chen T.F., Xiao W., Shang Q., Qin J.P., Wang Z.Z., 2011. HPLC Determination of Luteolin in *Eclipta prostrata* from Different Producing Area. Journal of Nanjing University of Traditional Chinese Medicine 2, 158-160. |

**Table S2: The 828 significant genes associated with hypertension**

| **Gene Symbol** | **Uniprot ID** | **Gene Symbol** | **Uniprot ID** | **Gene Symbol** | **Uniprot ID** |
| --- | --- | --- | --- | --- | --- |
| AGT | P01019 | GNAI1 | P63096 | GABBR1 | Q9UBS5 |
| INS | P01308 | KNG1 | P01042 | HAO2 | Q9NYQ3 |
| ACE | P12821 | GHRH | P01286 | PLAUR | Q03405 |
| REN | P00797 | FAS | P25445 | ATP1A2 | P50993 |
| EDN1 | P05305 | MAPKAPK2 | P49137 | PHEX | P78562 |
| LEP | P41159 | C5AR1 | P21730 | CACNB2 | Q08289 |
| NOS3 | P29474 | BCL2 | P10415 | PLCD3 | Q8N3E9 |
| ADD1 | P35611 | CYP1B1 | Q16678 | CSK | P41240 |
| NPPA | P01160 | KCNJ1 | P48048 | THBS4 | P35443 |
| CYP11B2 | P19099 | JAK1 | P23458 | EP300 | Q09472 |
| ANG | P03950 | GAPDH | P04406 | MAPT | P10636 |
| ADRB2 | P07550 | AQP1 | P29972 | SUCNR1 | Q9BXA5 |
| AGTR1 | P30556 | LPIN1 | Q14693 | TRPM6 | Q9BX84 |
| ADIPOQ | Q15848 | TGFBR1 | P36897 | CP | P00450 |
| TNF | P01375 | PRKG1 | Q13976 | COL1A2 | P08123 |
| RHOD | O00212 | LIPC | P11150 | RCAN1 | P53805 |
| NR3C2 | P08235 | HGF | P14210 | MAPK9 | P45984 |
| IL6 | P05231 | DDAH1 | O94760 | WISP1 | O95388 |
| PPARG | P37231 | JAK2 | O60674 | MRC1 | P22897 |
| BMPR2 | Q13873 | OLR1 | P78380 | COX4NB | O43402 |
| EDNRA | P25101 | IL17A | Q16552 | PRKCG | P05129 |
| WNK4 | Q96J92 | ATP2A2 | P16615 | CDKN1A | P38936 |
| CRP | P02741 | AHR | P35869 | PTPN1 | P18031 |
| HBB | P68871 | PRCP | P42785 | IL1R1 | P14778 |
| SELP | P16109 | NPY1R | P25929 | KLK11 | Q9UBX7 |
| RHOA | P61586 | CFH | P08603 | SMAD1 | Q15797 |
| SERPINA4 | P29622 | RENBP | P51606 | CACNA1D | Q01668 |
| SERPINE1 | P05121 | ERAP2 | Q6P179 | CASP8 | Q14790 |
| HMOX1 | P09601 | CLU | P10909 | ADORA1 | P30542 |
| HRAS | P01112 | ARL6IP5 | O75915 | QPCT | Q16769 |
| ACE2 | Q9BYF1 | IL6ST | P40189 | CMYA5 | Q8N3K9 |
| EPO | P01588 | COL4A3 | Q01955 | ENG | P17813 |
| WNK1 | Q9H4A3 | LGALS3 | P17931 | A1BG | P04217 |
| PTGS2 | P35354 | SLC12A6 | Q9UHW9 | ABCG8 | Q9H221 |
| NOS2 | P35228 | FGF2 | P09038 | ROS1 | P08922 |
| VEGFA | P15692 | TRHR | P34981 | TNFRSF10A | O00220 |
| F2 | P00734 | IFNG | P01579 | HPCAL1 | P37235 |
| HSD11B2 | P80365 | BMP2 | P12643 | NPHS2 | Q9NP85 |
| LPA | P08519 | APEX1 | P27695 | PREP | P48147 |
| APOE | P02649 | RHO | P08100 | DMD | P11532 |
| SGK1 | O00141 | S100A4 | P26447 | S1PR2 | O95136 |
| MAT2B | Q9NZL9 | MKKS | Q9NPJ1 | SLC34A2 | O95436 |
| ACSM3 | Q53FZ2 | ROCK2 | O75116 | CYSLTR2 | Q9NS75 |
| IGF1 | P05019 | TLR4 | O00206 | MMP7 | P09237 |
| ACTG2 | P63267 | RTN4 | Q9NQC3 | HOXA5 | P20719 |
| ADM | P35318 | SLC2A1 | P11166 | IL1RN | P18510 |
| APOB | P04114 | TGFB3 | P10600 | THRA | P10827 |
| GNB3 | P16520 | MTR | Q99707 | HLA-A | P04439 |
| VWF | P04275 | IGF1R | P08069 | AXL | P30530 |
| MMP9 | P14780 | CACNA1C | Q13936 | RFFL | Q8WZ73 |
| PPARA | Q07869 | ATP1B1 | P05026 | ARRDC3 | Q96B67 |
| POMC | P01189 | TACR1 | P25103 | ABO | P16442 |
| PTH | P01270 | IL2RA | P01589 | GSTM5 | P46439 |
| ADRA2B | P18089 | TBXAS1 | P24557 | NPY5R | Q15761 |
| ICAM1 | P05362 | ATP6AP2 | O75787 | FGFBP1 | Q14512 |
| NOS1 | P29475 | PROS1 | P07225 | GJA4 | P35212 |
| LPL | P06858 | F11R | Q9Y624 | CACNA1H | O95180 |
| EDNRB | P24530 | IL8 | P10145 | DRD3 | P35462 |
| PPP3CA | Q08209 | HSF1 | Q00613 | AVPR2 | P30518 |
| AKT1 | P31749 | SRC | P12931 | PDGFD | Q9GZP0 |
| PRL | P01236 | PFN1 | P07737 | G6PD | P11413 |
| CAT | P04040 | ADRA1A | P35348 | FSHR | P23945 |
| CAV1 | Q03135 | HLA-DQB1 | P01920 | SGPL1 | O95470 |
| GHRL | Q9UBU3 | CPB2 | Q96IY4 | IGFBP1 | P08833 |
| PRKCA | P17252 | SULT1A1 | P50225 | HSPB7 | Q9UBY9 |
| ADRB3 | P13945 | RLN1 | P04808 | TGFA | P01135 |
| INSR | P06213 | PRSS8 | Q16651 | CYP2C19 | P33261 |
| NEDD4L | Q96PU5 | CYP2C9 | P11712 | SORBS1 | Q9BX66 |
| NFKB1 | P19838 | ELANE | P08246 | MGAM | O43451 |
| FOS | P01100 | GSTM3 | P21266 | ADM2 | Q7Z4H4 |
| NPPB | P16860 | GATA6 | Q92908 | MMP8 | P22894 |
| GJA5 | P36382 | NEDD4 | P46934 | PRKAA1 | Q13131 |
| IL1B | P01584 | COX1 | P00395 | TLX2 | O43763 |
| SLC6A4 | P31645 | WNK2 | Q9Y3S1 | GRK1 | Q15835 |
| HLA-DOA | P06340 | MAOB | P27338 | ABCA3 | Q99758 |
| THBD | P07204 | MYC | P01106 | CYP2C8 | P10632 |
| SLC12A1 | Q13621 | HLA-DQA2 | P01906 | ATP8 | P03928 |
| ADRB1 | P08588 | S100A6 | P06703 | TG | P01266 |
| VCAM1 | P19320 | MCAT | Q8IVS2 | ITGA4 | P13612 |
| RGS2 | P41220 | KCNN4 | O15554 | SLC14A2 | Q15849 |
| AVP | P01185 | XYLT2 | Q9H1B5 | PDE3A | Q14432 |
| APLN | Q9ULZ1 | PPIG | Q13427 | LDOC1 | O95751 |
| CYP3A5 | P20815 | NFATC2 | Q13469 | PAPSS1 | O43252 |
| ELN | P15502 | SIRT1 | Q96EB6 | ALOX15 | P16050 |
| CALCA | P06881 | ITGAL | P20701 | PDIK1L | Q8N165 |
| GRK4 | P32298 | APLNR | P35414 | NPCDR1 | Q9BY65 |
| ROCK1 | Q13464 | CD63 | P08962 | RPS6KA3 | P51812 |
| CST3 | P01034 | HNF1A | P20823 | SLC4A4 | Q9Y6R1 |
| SCNN1B | P51168 | PHB2 | Q99623 | RARRES2 | Q99969 |
| EDN3 | P14138 | KCNMB4 | Q86W47 | SMAD7 | O15105 |
| HIF1A | Q16665 | PAH | P00439 | PDE4D | Q08499 |
| ANGPT1 | Q15389 | HSPD1 | P10809 | ARHGEF12 | Q9NZN5 |
| TH | P07101 | MAPK3 | P27361 | HSPA8 | P11142 |
| ADRA2A | P08913 | TNC | P24821 | CEBPZ | Q03701 |
| DDAH2 | O95865 | GHR | P10912 | BLK | P51451 |
| MTHFR | P42898 | HLA-DPB1 | P04440 | DNASE1 | P24855 |
| HSPA4 | P34932 | STAT1 | P42224 | SLC4A2 | P04920 |
| CD36 | P16671 | AQP2 | P41181 | TRPC6 | Q9Y210 |
| IAPP | P10997 | SLC5A1 | P13866 | HRH2 | P25021 |
| EGFR | P00533 | NPY6R | Q99463 | CALCR | P30988 |
| C3 | P01024 | GUCA2B | Q16661 | GJB1 | P08034 |
| MMP2 | P08253 | DEFA1 | P59665 | CDH13 | P55290 |
| SLC9A1 | P19634 | KCNMB1 | Q16558 | SLC22A3 | O75751 |
| UTS2 | O95399 | CYP27B1 | O15528 | CCR7 | P32248 |
| ATP1A1 | P05023 | ID1 | P41134 | SMAD2 | Q15796 |
| GCG | P01275 | ADD3 | Q9UEY8 | DNTT | P04053 |
| HSD11B1 | P28845 | IGKV1D-39 | P04432 | GYS1 | P13807 |
| CMA1 | P23946 | GLO1 | Q04760 | PLAU | P00749 |
| NGF | P01138 | P2RY2 | P41231 | PTPRO | Q16827 |
| ESR1 | P03372 | PROK1 | P58294 | IGF2 | P01344 |
| HP | P00738 | EPAS1 | Q99814 | DPP4 | P27487 |
| ESR2 | Q92731 | HMOX2 | P30519 | SERPINB2 | P05120 |
| AGTR2 | P50052 | SERPINA3 | P01011 | PSMA6 | P60900 |
| SOD1 | P00441 | IL11 | P20809 | TNFRSF11B | O00300 |
| EPHX2 | P34913 | KLK1 | P06870 | XDH | P47989 |
| MTPN | P58546 | ADRA2C | P18825 | CYP11A1 | P05108 |
| GCLC | P48506 | PCNA | P12004 | STIM1 | Q13586 |
| CCL2 | P13500 | TBXA2R | P21731 | ILK | Q13418 |
| RAC1 | P63000 | FASLG | P48023 | CDH15 | P55291 |
| CYBA | P13498 | SLC26A6 | Q9BXS9 | CCR5 | P51681 |
| JUN | P05412 | G6PC | P35575 | CSMD1 | Q96PZ7 |
| LDLR | P01130 | ZNF652 | Q9Y2D9 | SLC6A9 | P48067 |
| COG2 | Q14746 | HSD3B1 | P14060 | HFE | Q30201 |
| CALM1 | P0DP23 | ANPEP | P15144 | CABIN1 | Q9Y6J0 |
| AR | P10275 | PRAM1 | Q96QH2 | ACADSB | P45954 |
| MC3R | P41968 | LMNA | P02545 | SMURF1 | Q9HCE7 |
| SOD3 | P08294 | YEATS4 | O95619 | PPP1R12A | O14974 |
| LEPR | P48357 | IL13RA2 | Q14627 | NDUFC2 | O95298 |
| GSTM1 | P09488 | ALOX5 | P09917 | IL1A | P01583 |
| GCA | P28676 | TNFRSF4 | P43489 | PLA2G1B | P04054 |
| TNFRSF1B | P20333 | TXNL4B | Q9NX01 | ARAP1 | Q96P48 |
| KCNJ11 | Q14654 | KCNK2 | O95069 | BRIP1 | Q9BX63 |
| SST | P61278 | IL7 | P13232 | NR1H4 | Q96RI1 |
| PPARGC1A | Q9UBK2 | CXCR2 | P25025 | MPV17 | P39210 |
| RETN | Q9HD89 | IL1F8 | Q9NZH7 | IKBKB | O14920 |
| ALB | P02768 | KYNU | Q16719 | KCNE1 | P15382 |
| BDKRB2 | P30411 | SP2 | Q02086 | VAV2 | P52735 |
| STK39 | Q9UEW8 | SOAT2 | O75908 | UCP1 | P25874 |
| NR3C1 | P04150 | PRKAA2 | P54646 | SERPINE2 | P07093 |
| DBH | P09172 | CTLA4 | P16410 | ISYNA1 | Q9NPH2 |
| CRH | P06850 | CDKN2B | P42772 | MAP1LC3B | Q9GZQ8 |
| CYP2D6 | P10635 | IL4 | P05112 | CRHR2 | Q13324 |
| NPR3 | P17342 | GPR98 | Q8WXG9 | SLC12A2 | P55011 |
| CYP11B1 | P15538 | CCND1 | P24385 | KCNB1 | Q14721 |
| NPY | P01303 | RAMP1 | O60894 | CYP21A2 | P08686 |
| CYBB | P04839 | HNRNPAB | Q99729 | CXCL10 | P02778 |
| CHGA | P10645 | GPA33 | Q99795 | ERI1 | Q8IV48 |
| ANGPT2 | O15123 | DDIT3 | P35638 | PDGFRB | P09619 |
| SOD2 | P04179 | NBN | O60934 | KISS1 | Q15726 |
| GATA4 | P43694 | HCRT | O43612 | BBS4 | Q96RK4 |
| SCNN1A | P37088 | GCH1 | P30793 | ATF1 | P18846 |
| KDR | P35968 | DRD4 | P21917 | GRP | P07492 |
| SCNN1G | P51170 | CYP3A7 | P24462 | GLUL | P15104 |
| MYH9 | P35579 | PROM1 | O43490 | FABP3 | P05413 |
| HMGCR | P04035 | FABP6 | P51161 | SELE | P16581 |
| PNMT | P11086 | HLA-DQA1 | P01909 | PIM1 | P11309 |
| PKD1 | P98161 | FLT3LG | P49771 | EGF | P01133 |
| BMP6 | P22004 | LINGO1 | Q96FE5 | CARTPT | Q16568 |
| PDE5A | O76074 | AQP4 | P55087 | EGLN3 | Q9H6Z9 |
| CYP2J2 | P51589 | PDC | P20941 | DCP2 | Q8IU60 |
| GJA1 | P17302 | ABCA1 | O95477 | IKBKAP | O95163 |
| GSTT1 | P30711 | SCG2 | P13521 | KCNN3 | Q9UGI6 |
| PTGS1 | P23219 | E2F2 | Q14209 | TXN2 | Q99757 |
| CD4 | P01730 | FURIN | P09958 | TPH1 | P17752 |
| CASP3 | P42574 | AHSG | P02765 | UCP3 | P55916 |
| ACVRL1 | P37023 | PGD | P52209 | CTGF | P29279 |
| PLAT | P00750 | RAMP2 | O60895 | PAG1 | Q9NWQ8 |
| CLCNKB | P51801 | BCR | P11274 | CAMK2G | Q13555 |
| ALDH2 | P05091 | LPCAT3 | Q6P1A2 | IL2 | P60568 |
| HTR2A | P28223 | RAF1 | P04049 | PLD2 | O14939 |
| CCL5 | P13501 | SLC9A3R1 | O14745 | CDK5R1 | Q15078 |
| SLC2A4 | P14672 | CPE | P16870 | TNNI3 | P19429 |
| ADRBK1 | P25098 | PTGIR | P43119 | SLC17A5 | Q9NRA2 |
| IRS1 | P35568 | IL18 | Q14116 | TAP1 | Q03518 |
| SLC26A4 | O43511 | LMX1B | O60663 | PPP1R14A | Q96A00 |
| TGFB1 | P01137 | F3 | P13726 | IL13 | P35225 |
| MAPK14 | Q16539 | PPY | P01298 | NF1 | P21359 |
| UCP2 | P55851 | GABRA6 | Q16445 | BTN2A1 | Q7KYR7 |
| ADRA1B | P35368 | SLC9A3R2 | Q15599 | TFRC | P02786 |
| VIP | P01282 | FKBP1A | P62942 | TAC1 | P20366 |
| CTF1 | Q16619 | SLC2A9 | Q9NRM0 | PEPD | P12955 |
| MMP1 | P03956 | CAV2 | P51636 | SP1 | P08047 |
| GH1 | P01241 | RET | P07949 | SPANXC | Q9NY87 |
| APOA1 | P02647 | PDPK1 | O15530 | TRPC5 | Q9UL62 |
| BMP1 | P13497 | CXCR1 | P25024 | SLC16A1 | P53985 |
| SLC8A1 | P32418 | CYP1A1 | P04798 | PAPPA | Q13219 |
| NPR1 | P16066 | USP1 | O94782 | PRDX5 | P30044 |
| CYP4A11 | Q02928 | THBS1 | P07996 | NR1I2 | O75469 |
| CYP3A4 | P08684 | APOL1 | O14791 | ANXA1 | P04083 |
| SLC2A5 | P22732 | POU5F1 | Q01860 | GPR25 | O00155 |
| ABCB1 | P08183 | ABCG5 | Q9H222 | MYOC | Q99972 |
| NCF1 | P14598 | MEF2A | Q02078 | MAS1 | P04201 |
| FANCB | Q8NB91 | CXCR4 | P61073 | HPGD | P15428 |
| C2 | P06681 | EGR1 | P18146 | CASP9 | P55211 |
| HBA1 | P69905 | TGM2 | P21980 | CXCL12 | P48061 |
| CAPN10 | Q9HC96 | GNB2L1 | P63244 | ACAT1 | P24752 |
| CD34 | P28906 | GNG2 | P59768 | COL4A1 | P02462 |
| PPBP | P02775 | CKS1B | P61024 | PTEN | P60484 |
| KCNA5 | P22460 | PON2 | Q15165 | CD200 | P41217 |
| HTR1A | P08908 | CHEK2 | O96017 | PDYN | P01213 |
| NPHS1 | O60500 | CD8A | P01732 | MAT1A | Q00266 |
| ITGAM | P11215 | VHL | P40337 | PLEKHA7 | Q6IQ23 |
| ADCY10 | Q96PN6 | GDNF | P39905 | CTSB | P07858 |
| GNAS | P63092 | HSPB2 | Q16082 | HTR2B | P41595 |
| HLA-DRB1 | P01911 | UCN | P55089 | CD59 | P13987 |
| NOX1 | Q9Y5S8 | GATA5 | Q9BWX5 | ADAMTS13 | Q76LX8 |
| CDKN2A | P42771 | SLC2A2 | P11168 | NR4A2 | P43354 |
| SLC12A3 | P55017 | AVPR1A | P37288 | PARK2 | O60260 |
| CAST | P20810 | ADRBK2 | P35626 | NDFIP2 | Q9NV92 |
| ADRA1D | P25100 | SMTN | P53814 | GDF15 | Q99988 |
| CYP4F2 | P78329 | HDLBP | Q00341 | WT1 | P19544 |
| KLKB1 | P03952 | SPAG5 | Q96R06 | ACSM1 | Q08AH1 |
| INPPL1 | O15357 | ADH5 | P11766 | ILF3 | Q12906 |
| F5 | P12259 | BNIP1 | Q12981 | ACHE | P22303 |
| TXK | P42681 | NEUROD1 | Q13562 | MTMR9 | Q96QG7 |
| PLA2G7 | Q13093 | CD19 | P15391 | CLOCK | O15516 |
| GC | P02774 | GNA12 | Q03113 | VNN1 | O95497 |
| KCNQ1 | P51787 | NPY2R | P49146 | SLC4A1 | P02730 |
| GRK5 | P34947 | HDAC2 | Q92769 | SDK1 | Q7Z5N4 |
| STAT3 | P40763 | IFNA1 | P01562 | PTHLH | P12272 |
| NOX4 | Q9NPH5 | TGFBR2 | P37173 | MEOX2 | P50222 |
| HSPA1A | P0DMV8 | FAAH | O00519 | PIK3R1 | P27986 |
| SRY | Q05066 | SELL | P14151 | PPP3R1 | P63098 |
| PLCD1 | P51178 | NT5E | P21589 | TPH2 | Q8IWU9 |
| HSP90AA1 | P07900 | CA2 | P00918 | ALPP | P05187 |
| UMOD | P07911 | CYR61 | O00622 | ALOX12 | P18054 |
| PENK | P01210 | PSMB9 | P28065 | CXCL5 | P42830 |
| KITLG | P21583 | NPPC | P23582 | LNPEP | Q9UIQ6 |
| GPX1 | P07203 | APOBEC3F | Q8IUX4 | CREBBP | Q92793 |
| SLC22A2 | O15244 | EGLN1 | Q9GZT9 | ACTN2 | P35609 |
| SLC9A3 | P48764 | MAP2K1 | Q02750 | ACP1 | P24666 |
| DRD1 | P21728 | PLG | P00747 | GNA14 | O95837 |
| GCGR | P47871 | ALOX5AP | P20292 | KCNJ10 | P78508 |
| TIMP1 | P01033 | S100A8 | P05109 | FABP2 | P12104 |
| TIMP2 | P16035 | RETNLB | Q9BQ08 | HLA-B | P01889 |
| PDGFB | P01127 | MFN2 | O95140 | IL13RA1 | P78552 |
| RGS5 | O15539 | MARK2 | Q7KZI7 | CYP1A2 | P05177 |
| ECE1 | P42892 | ITGB1BP2 | Q9UKP3 | NOTCH4 | Q99466 |
| FBN1 | P35555 | TXN | P10599 | SCN2A | Q99250 |
| B2M | P61769 | AMY1A | P04745 | PARP1 | P09874 |
| GAL | P22466 | MC4R | P32245 | DNAJB11 | Q9UBS4 |
| SLC6A2 | P23975 | APOC3 | P02656 | TRPM4 | Q8TD43 |
| IL10 | P22301 | PAK1 | Q13153 | SCN7A | Q01118 |
| PON1 | P27169 | XYLT1 | Q86Y38 | CLCN3 | P51790 |
| TRH | P20396 | VDR | P11473 | T | O15178 |
| EDN2 | P20800 | CYP7A1 | P22680 | CETP | P11597 |
| ATP2B1 | P20020 | GGT1 | P19440 | FMO3 | P31513 |
| ADD2 | P35612 | CD2 | P06729 | NCAM1 | P13591 |
| ITGA2 | P17301 | SF1 | Q15637 | RFC2 | P35250 |
| ADORA2A | P29274 | USP2 | O75604 | SLC24A4 | Q8NFF2 |
| PROC | P04070 | MMP3 | P08254 | FUT1 | P19526 |
| OXT | P01178 | GTPBP4 | Q9BZE4 | EPHX1 | P07099 |
| S100A1 | P23297 | SUCLG2 | Q96I99 | FN1 | P02751 |
| C4A | P0C0L4 | TBK1 | Q9UHD2 | CCNA2 | P20248 |
| NFATC3 | Q12968 | DSP | P15924 | DCN | P07585 |
| CYP17A1 | P05093 | PECAM1 | P16284 | VIM | P08670 |
| MYOCD | Q8IZQ8 | TF | P02787 | NTRK1 | P04629 |
| ATP5G1 | P05496 | BDKRB1 | P46663 | SLC4A8 | Q2Y0W8 |
| DRD2 | P14416 | ATXN3 | P54252 | CDH5 | P33151 |
| FGF5 | P12034 | GRK6 | P43250 | PCSK6 | P29122 |
| AHSP | Q9NZD4 | SHC1 | P29353 | NFKBIB | Q15653 |
| RNLS | Q5VYX0 | CRY1 | Q16526 | CCL23 | P55773 |
| SERPINA1 | P01009 | S1PR1 | P21453 | GSK3B | P49841 |
| SPP1 | P10451 | BCL2L1 | Q07817 | PRRX1 | P54821 |
| TRPV1 | Q8NER1 | ATP6V1B1 | P15313 | MSR1 | P21757 |
| PTGIS | Q16647 | ARHGEF1 | Q92888 | RABGAP1L | Q5R372 |
| EMILIN1 | Q9Y6C2 | PRDX3 | P30048 | GJC1 | P36383 |
| MAPK1 | P28482 | IL15 | P40933 | ANKRD1 | Q15327 |
| CORIN | Q9Y5Q5 | RGS20 | O76081 | SERPINA6 | P08185 |
| SMAD3 | P84022 | GLI1 | P08151 | SLC22A6 | Q4U2R8 |
| VEGFB | P49765 | RAMP3 | O60896 | IGFBP2 | P18065 |
| CYP19A1 | P11511 | COMT | P21964 | MUC5AC | P98088 |
| ATP6V0A1 | Q93050 | EPOR | P19235 | TBRG1 | Q3YBR2 |
| GORASP1 | Q9BQQ3 | MMP14 | P50281 | YY1 | P25490 |
| BMP4 | P12644 | LGALS2 | P05162 | ENPP1 | P22413 |
| CX3CL1 | P78423 | RYR2 | Q92736 | IER3 | P46695 |

**Table S3: Docking results of 10 active ingredients and NOS3**

| **Ingredients** | **Total_Score** | **Crash** | **Polar** |
| --- | --- | --- | --- |
| Histidine | 6.3348 | -0.1440 | 8.3102 |
| Cedar acid | 6.2446 | -0.4673 | 4.5611 |
| Linalool | 6.1754 | -0.9512 | 1.1426 |
| p-Hydroxybenzoic acid | 6.0571 | -0.4822 | 3.8879 |
| Shikimic acid | 4.6199 | -0.6389 | 3.5727 |
| Salicylic acid | 4.5084 | -0.2145 | 5.6569 |
| Nicotinic acid | 4.4987 | -0.2532 | 4.4976 |
| pulegone | 4.4954 | -0.5670 | 0.0000 |
| 5-Hydroxymethylfurfural | 4.2945 | -0.3198 | 2.4507 |
| L-Bornyl acetate | 4.2346 | -0.3992 | 1.7464 |

Total score indicated the total dock score.

Crash-score reveals the ability of a compound to penetrate a protein active site.

Polar score: the polar interaction between the protein and ligand.

**Table S4: Docking results of 10 active ingredients and SRC**

| **Ingredients** | **Total_Score** | **Crash** | **Polar** |
| --- | --- | --- | --- |
| Shikimic acid | 8.2297 | -0.7020 | 7.4211 |
| Salicylic acid | 8.2043 | -0.5401 | 6.9977 |
| Histidine | 7.7734 | -0.4526 | 7.6186 |
| Cedar acid | 6.8966 | -0.9119 | 4.4851 |
| Nicotinic acid | 6,7675 | -0.0813 | 6.1328 |
| p-Hydroxybenzoic acid | 6.1640 | -1.0627 | 6.7862 |
| Linalool | 5.1493 | -0.7765 | 1.3457 |
| pulegone | 4.5547 | -1.4575 | 0.5330 |
| 5-Hydroxymethylfurfural | 4.3716 | -0.3800 | 4.4570 |
| L-Bornyl acetate | 2.9957 | -0.4851 | 1.2889 |

Total score indicated the total dock score.

Crash-score reveals the ability of a compound to penetrate a protein active site.

Polar score: the polar interaction between the protein and ligand.

**Table S5: Docking results of 10 active ingredients and PI3K**

| **Ingredients** | **Total_Score** | **Crash** | **Polar** |
| --- | --- | --- | --- |
| Cedar acid | 7.2253 | -1.0899 | 5.3711 |
| p-Hydroxybenzoic acid | 6.5506 | -0.6399 | 4.3699 |
| Linalool | 6.1761 | -1.3432 | 1.9788 |
| Salicylic acid | 5.6957 | -0.1617 | 4.2707 |
| Histidine | 5.3442 | -0.6108 | 4.1117 |
| Nicotinic acid | 5.0384 | -0.1363 | 3.1245 |
| Shikimic acid | 4.7101 | -1.6518 | 4.1782 |
| 5-Hydroxymethylfurfural | 4.4065 | -0.3841 | 2.2528 |
| pulegone | 3.2292 | -3.0044 | 0.8943 |
| L-Bornyl acetate | 2.2141 | -0.2124 | 1.2882 |

Total score indicated the total dock score.

Crash-score reveals the ability of a compound to penetrate a protein active site.

Polar score: the polar interaction between the protein and ligand.

**Table S6: Docking results of 10 active ingredients and AKT**

| **Ingredients** | **Total_Score** | **Crash** | **Polar** |
| --- | --- | --- | --- |
| Cedar acid | 8.2980 | -0.6007 | 8.1101 |
| Shikimic acid | 7.2071 | -0.8317 | 8.7737 |
| Nicotinic acid | 7.1323 | -0.2939 | 7.2074 |
| Salicylic acid | 5.4530 | -0.2868 | 6.4013 |
| Histidine | 4.7987 | -0.1960 | 6.0716 |
| Linalool | 4.5285 | -0.8561 | 1.7543 |
| p-Hydroxybenzoic acid | 4.3329 | -0.1517 | 5.5838 |
| 5-Hydroxymethylfurfural | 3.7862 | -0.2860 | 4.2612 |
| L-Bornyl acetate | 3.3792 | -0.1780 | 2.1381 |
| pulegone | 3.1018 | -0.4775 | 2.3304 |

Total score indicated the total dock score.

Crash-score reveals the ability of a compound to penetrate a protein active site.

Polar score: the polar interaction between the protein and ligand.
