# Supplementary material for: A network pharmacology-based approach to explore the active ingredients and molecular mechanism of Lei-gong-gen formula granule on a spontaneously hypertensive rat model
Source: Chin Med. 2021 Oct 9;16:99. doi: 10.1186/s13020-021-00507-1 (PMC8501634; doi:10.1186/s13020-021-00507-1)
Supplement: Supplementary file 2 — Additional file 2: Figures S1-S18. The mass spectrum comparison of components in LFG with the reference standard. [file 13020_2021_507_MOESM2_ESM.docx]

A

B

C

D

**Figure S1: Shikimic Acid negative ion mode mass spectrum. Primary mass spectrum of (A) shikimic acid standards and (B) samples; Secondary mass spectrum of (C) shikimic acid standards and (D) samples.**

A

B

C

D

**Figure S2: Nicotinic acid positive ion mode mass spectrum. Primary mass spectrum of (A) nicotinic acid standards and (B) samples; Secondary mass spectrum of (C) nicotinic acid standards and (D) samples.**

A

B

C

D

**Figure S3: p-Hydroxybenzoic acid negative ion mode mass spectrum. Primary mass spectrum of (A) p-hydroxybenzoic acid standards and (B) samples; Secondary mass spectrum of (C) p-hydroxybenzoic acid standards and (D) samples.**

A

B

C

D

**Figure S4: Chlorogenic acid negative ion mode mass spectrum. Primary mass spectrum of (A) chlorogenic acid standards and (B) samples; Secondary mass spectrum of (C) chlorogenic acid standards and (D) samples.**

A

B

C

D

**Figure S5: 4-Dicaffeoylquinic acid negative ion mode mass spectrum. Primary mass spectrum of (A) 4-dicaffeoylquinic acid standards and (B) samples; Secondary mass spectrum of (C) 4-dicaffeoylquinic acid standards and (D) samples.**

A

B

C

D

**Figure S6: Isochlorogenic acid C negative ion mode mass spectrum. Primary mass spectrum of (A) isochlorogenic acid C standards and (B) samples; Secondary mass spectrum of (C) isochlorogenic acid C standards and (D) samples.**

A

B

C

D

**Figure S7: Ferulic acid negative ion mode mass spectrum. Primary mass spectrum of (A) ferulic acid standards and (B) samples; Secondary mass spectrum of (C) ferulic acid standards and (D) samples.**

A

B

C

D

**Figure S8: Neoastilbin negative ion mode mass spectrum. Primary mass spectrum of (A) neoastilbin standards and (B) samples; Secondary mass spectrum of (C) neoastilbin standards and (D) samples.**

A

B

C

D

**Figure S9: Astilbin negative ion mode mass spectrum. Primary mass spectrum of (A) astilbin standards and (B) samples; Secondary mass spectrum of (C) astilbin standards and (D) samples.**

A

B

C

D

**Figure S10: Neoisoastilbin negative ion mode mass spectrum. Primary mass spectrum of (A) neoisoastilbin standards and (B) samples; Secondary mass spectrum of (C) neoisoastilbin standards and (D) samples.**

A

B

C

D

**Figure S11: Isoastilbin negative ion mode mass spectrum. Primary mass spectrum of (A) isoastilbin standards and (B) samples; Secondary mass spectrum of (C) isoastilbin standards and (D) samples.**

A

B

D

C

**Figure S12: Engeletin negative ion mode mass spectrum. Primary mass spectrum of (A) engeletin standards and (B) samples; Secondary mass spectrum of (C) engeletin standards and (D) samples.**

A

B

C

D

**Figure S13: Isoengelitin negative ion mode mass spectrum. Primary mass spectrum of (A) isoengelitin standards and (B) samples; Secondary mass spectrum of (C) isoengelitin standards and (D) samples.**

A

B

C

D

**Figure S14: Salicylic acid negative ion mode mass spectrum. Primary mass spectrum of (A) salicylic acid standards and (B) samples; Secondary mass spectrum of (C) salicylic acid standards and (D) samples.**

A

B

C

D

**Figure S15: luteolin negative ion mode mass spectrum. Primary mass spectrum of (A) luteolin standards and (B) samples; Secondary mass spectrum of (C) luteolin standards and (D) samples.**

A

B

**Figure S16: Wedelolactone negative ion mode mass spectrum. Primary mass spectrum of (A) samples; Secondary mass spectrum of (B) samples.**

A

B

**Figure S17: Madecassic acid negative ion mode mass spectrum. Primary mass spectrum of (A) samples; Secondary mass spectrum of (B) samples.**

A

B

**Figure S18: Asiatic acid negative ion mode mass spectrum. Primary mass spectrum of (A) samples; Secondary mass spectrum of (B) samples.**
